# Islet function impairment outcomes of immune checkpoint inhibitors in cancer patients: a systematic review and meta-analysis

**DOI:** 10.3389/fimmu.2026.1669492

**Published:** 2026-03-19

**Authors:** Qi Hu, Yongzheng Fan, Ping He

**Affiliations:** 1 Department of Pharmacy, Union Hospital, Tongji Medical College, Huazhong University of Science and Technology, Wuhan, Hubei, China; 2 Department of Pharmacy, The 991th Hospital of Joint Logistic Support Force of People’s Liberation Army, Xiangyang, Hubei, China; 3 Department of Geriatrics, Union Hospital, Tongji Medical College, Huazhong University of Science and Technology, Wuhan, Hubei, China

**Keywords:** CTLA-4 inhibitors, diabetes mellitus, endocrine adverse events, immune checkpoint inhibitors, PD-1 inhibitors, PD-L1 inhibitors

## Abstract

**Background:**

Immune checkpoint inhibitors (ICPis) are associated with islet function impairment (IFI), manifesting as hyperglycemia, diabetes mellitus (DM), or diabetic ketoacidosis (DKA). Delayed detection and management may lead to irreversible β-cell damage and life-threatening complications. We conducted a systematic review and meta-analysis to assess the risk of IFI associated with ICPis.

**Methods:**

Following PICOS principles, we searched PubMed, Embase, Cochrane, CNKI, Wanfang, CBM, and VIP databases from inception to October 24, 2024. We included randomized controlled trials (RCTs) comparing ICPis versus non-ICPis regimens in cancer patients. Outcomes included hyperglycemia, DM, and DKA. Risk ratios (RRs) with 95% confidence intervals (CIs) were pooled using fixed- or random-effects models. Quality was assessed with the Cochrane Risk of Bias tool, and publication bias was evaluated by Begg’s test. The protocol was registered with PROSPERO (CRD42025639629).

**Results:**

A total of 31 RCT studies with 15,417 patients were included in this study. Results showed that ICPis treatment significantly increased the risk of associated IFI (RR = 1.30, 95%CI: 1.10-1.53, *P* = 0.002), the risk of grade 3-5 (RR = 2.20, 95%CI: 1.50-3.23, *P* < 0.0001) and type 1 diabetes (T1DM) (RR = 3.38, 95%CI: 1.66-6.88, *P* = 0.0008) compared to those treated with non-ICPis; Subgroup analysis showed that, compared with non-ICPis, the PD-1 inhibitor and Pembrolizumab groups significantly increased the incidence of developing IFI (RR = 1.57, 95%CI: 1.22-2.01, *P* = 0.0005; RR = 2.38, 95%CI: 1.43-3.97, *P* = 0.0009); Patients with NSCLC receiving ICPis had a significantly higher risk of developing IFI compared with non-ICPis (RR = 1.32, 95%CI: 1.01-1.72, *P* = 0.04). Compared to their respective non-ICPis controls, the point estimate for IFI risk was lower with ICPis plus chemotherapy (RR = 1.23) than with ICPis monotherapy (RR = 1.43); a similar pattern was observed for grade 3–5 IFI (RR = 1.53 vs. 3.39). No publication bias was detected.

**Conclusions:**

ICPis significantly increase the risk of IFI, particularly T1DM and severe (grade 3-5) events. PD-1 inhibitors and patients with NSCLC represent high-risk subgroups. We strongly recommend multidisciplinary monitoring and proactive blood glucose management.

**Systematic review registration:**

https://www.crd.york.ac.uk/PROSPERO/myprospero, identifier CRD42025639629

## 1 Introduction

Immune checkpoint inhibitors (ICPis) specifically block immunosuppressive molecules, target T cell regulatory pathways, enhance anti-tumor immune response, inhibit immune escape, and induce tumor cell death, thus achieving clinical anti-tumor therapeutic goals (1). Currently, ICPis include cytotoxic T lymphocyte-associated antigen-4 inhibitors (CTLA-4 inhibitors), programmed cell death protein 1, and programmed death-ligand 1inhibitors (PD-L1 inhibitors) (2). At present, the application of immunotherapy has received great attention, especially in the combination of immunotherapy with chemotherapy or radiotherapy, which is actively moving toward first-line treatment, and its therapeutic effect has brought benefits to the treatment of advanced and recurrent malignant tumors. Immunotherapy can significantly improve the prognosis and overall survival of many malignant tumors, including urothelial carcinoma, renal cell carcinoma, melanoma, non-small cell lung cancer, colorectal cancer, and Hodgkin’s lymphoma, as well as slow tumor progression (3–5).

With an in-depth study of the ICPis mechanism and its wide application in clinical practice, it has been confirmed that blocking ICPis in tumor therapy not only promotes T cell-mediated immune destruction of tumor cells, but may also promote autoimmune activities in different organs, including the skin, cardiovascular, musculoskeletal, liver, gastrointestinal, lung, and endocrine systems. Concomitant symptoms such as pneumonia, fatigue, rash, diarrhea, colitis, arthritis, hepatitis, hyperthyroidism, hypoadrenal function, and pancreatitis are often referred to as immune-related adverse events (3, 6, 7). Studies have shown that approximately 4-30% of patients develop endocrine disorders (8), ICPis-associated islet function impairment (IFI) is relatively rare, including hyperglycemia, DM and DKA, with an incidence of approximately 3.5% (9), However, most of them require lifelong insulin therapy, and the mechanism of action is unclear, which may be due to the activation of β-cell antigens (proinsulin and preinsulin antigens, tyrosine phosphatase-like insulinoma antigen, islet-specific glucose-6-phosphate protein, glutamate decarboxylase-65, zinc transporter 8, and islet amyloid polypeptide) by autologous active CD8+ T cells (10).

If ICPis-associated IFI is not detected and treated promptly, the disease can become severe and even lead to irreversible damage to β-cells, resulting in hyperglycemia, polydipsia, polyuria, and DKA, which can be life threatening and lead to death (11–13). According to the WHO Safety Reporting Database of Cases, there has been a significant increase in the number of people reporting ICPis-related IFI since 2017 (14). This has led to increased concern among endocrinologists and oncologists about whether ICPis therapy is associated with an increased risk of IFI in cancer patients. Several prior meta-analyses have investigated the association between ICPis and endocrine adverse events, including diabetes (11, 15–17). These studies provided foundational evidence but had certain limitations: some incorporated heterogeneous study designs (e.g., combining RCTs with observational studies and case reports) (11, 16, 18) which may introduce confounding; others focusing on RCTs (19, 20) were conducted before the completion of numerous recent, large-scale phase 3 trials (e.g., RATIONALE-303, ATALANTE/ENGOT-ov29) (21, 22) that have substantially expanded the ICPis landscape. Moreover, prior analyses lacked detailed subgroup comparisons across specific drug classes (PD-1 vs. PD-L1 vs. CTLA-4), individual tumor types, and treatment modalities (monotherapy vs. combination therapy) — gaps that the present study aims to fill. Crucially, granular analyses comparing risks among specific ICPis classes (PD-1 vs. PD-L1 vs. CTLA-4), across major tumor types, and between different therapeutic modalities (monotherapy versus combination regimens) remain insufficient. To address these gaps with the most contemporary and high-level evidence, we conducted this systematic review and meta-analysis exclusively of RCTs. Our primary objectives were: (1) to provide an updated and robust estimate of IFI risk; (2) to perform comprehensive subgroup analyses to identify high-risk scenarios; and (3) to evaluate the severity (grade 3-5) of ICPis-associated IFI. Our study aimed to provide comprehensive and systematic evidence-based medical guidance for ICPis-related IFI, a reference for toxicity management and safety guidance for rational clinical use of ICPis.

## 2 Methods

### 2.1 Agreement

This systematic review and meta-analysis set search criteria according to the principles of PICOS (patient population, interventions, controls, and outcome measures). We followed the Preferred Reporting Items for Systematic Reviews and Meta-Analyses (PRISMA) guidelines (23) (**Supplementary Table 1**) and registered in the international prospective registry of the systematic review PROSPERO (CRD42025639629).

### 2.2 Search strategy

We adopted a systematic search strategy. We searched PubMed, Embase, Cochrane, and other libraries as well as CNKI, Wanfang, CBM, VIP, and other electronic databases. The search began at the time of database construction and ended in October 2024. Two researchers independently screened all titles, abstracts, and full texts to determine whether they met the inclusion criteria, with a consensus reached through third-party adjudication in cases of disagreement. The search strategy is available in **Supplementary Table 2**.

### 2.3 Inclusion criteria

(1) Clearly diagnosed as a tumor, without limiting the type of tumor, regardless of whether basic treatment had been received; (2) included studies were RCTs for public publication; (3) clearly reported the number of IFI-related endocrine adverse reactions, such as hyperglycemia, T1DM, T2DM and DKA; (4) raw data are provided in the included studies, which are accessible and unadjusted; (5) the experimental group was treated with ICPis, and the control group was treated with chemoradiotherapy or placebo without ICPis (non-ICPis).

### 2.4 Exclusion criteria

(1) Animal, cell, and other basic research; (2) ICPis used in the control group; (3) reviews, case reports, meta-analyses, conference abstracts, systematic reviews, correspondence letters, plans, etc.; (4) incomplete data, inability to obtain full text and literature with a high risk of bias; (5) a study that determines whether it is a replication or the same population by trial registration number; the same data or clinical trial reports give priority to more complete or up-to-date study data.

### 2.5 Data extraction

Information extraction included author name, year of publication, trial characteristics (clinical trial NCT serial number, whether it was an international study, countries involved, first author, study location, study stage, tumor type, total number of patients, and treatment regimen), patient characteristics (sex, age, and outcome), intervention and control group size, and ICPis treatment dose. ICPis-related IFI outcomes included hyperglycemia, T2DM, DKA, and T1DM. According to the Common Terminology Criteria for Adverse Events (CTCAE), ICPis-related IFI were classified into grade 1-5 (24). In this study, grade 1–2 is defined as minor and grade 3–5 is defined as serious adverse events.

### 2.6 Quality assessment

The included studies were independently assessed using the Cochrane Bias Risk Tool. The quality of the clinical studies was evaluated in seven dimensions: random sequence generation (selection bias), allocation concealment (selection bias), intervention blinding (execution bias), outcome evaluation blinding (detection bias), incomplete outcome data (missing bias), selective reporting (reporting bias), and other bias. The study quality was divided into three levels: low risk of bias (+), high risk of bias (−), and unclear (?). Funnel plots were used to assess literature bias. A sensitivity analysis was used to determine whether the results were likely to be affected by a single study by deleting one study at a time. Stata 18.0 was used for sensitivity analysis, and publication bias was assessed by Begg’s test (*P*>0.05 indicating no publication bias).

### 2.7 Statistical analysis

The meta-analysis was performed using RevMan 5.4 and Stata18 software. All outcome indicators were categorized as categorical variables. Relative risk RR and 95% CI were used as effect size indicators, and the results were represented by forest plots. Each effect size was given a point estimate and 95% confidence interval (CI). Cochran’s Q test was used to evaluate inter-study heterogeneity, which was determined according to the size of *I^2^*. *A* fixed-effects model was used for analysis when *I*^2^ was ≤ 50%; otherwise, a random-effects model was used. *P* < 0.05 was considered statistically significant.

### 2.8 Quality of evidence

The overall quality of evidence was assessed using the Grading of Recommendations Assessment, Development, and Evaluation system (GRADE). The result was rated as high, moderate, low, and very low certainty of evidence. Including: study limitations, inconsistency, indirectness, imprecision, and publication bias. RCTs were considered high quality, and were downgraded to moderate, low, and very low one by one based on the rating content.

## 3 Results

### 3.1 Literature search results

**Figure 1:**
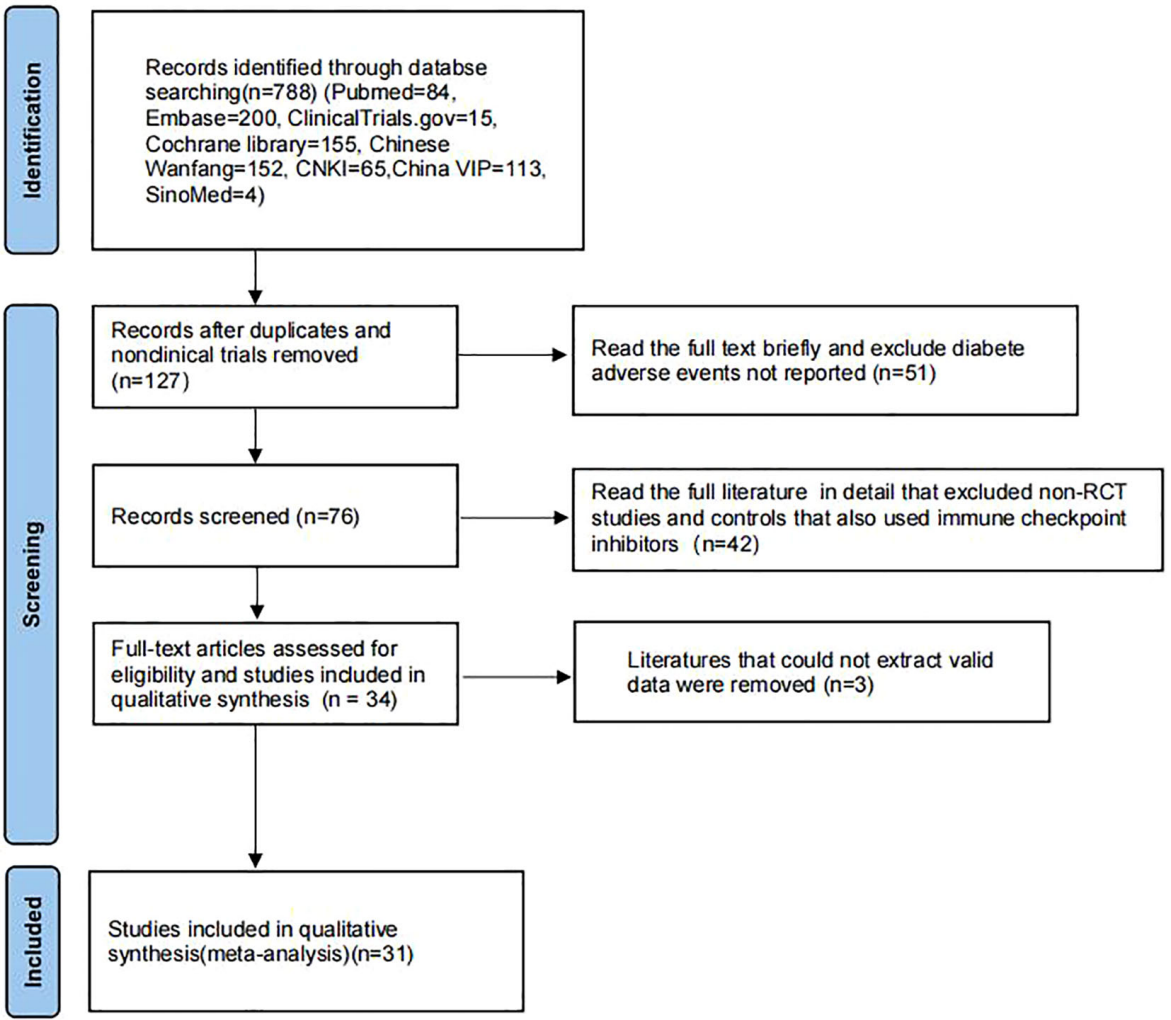
Flow diagram of study searching and selection process.

We searched PubMed, EMBASE, and Cochrane databases by combining subject terms and free words. By October 2024, 788 studies had been screened (**Figure 1**). After reading the titles and abstracts, 127 studies were retrieved by comparing and eliminating duplicate and non-clinical studies. After a brief reading of the full text, 51 unpublished diabetes-related adverse reactions were removed, followed by further detailed reading of the full text, which excluded 42 non-RCTs, and non-compliant studies in the control group. Three studies that could not extract effective data were excluded, and 31 studies with 15,417 patients were finally included in the meta-analysis.

### 3.2 Characteristics of the total study population

The included trials, conducted in 1 to 31 countries, had sample sizes ranging from 34 to 1286 (**Supplementary Table 3**) (21, 22, 25–53). There were 8 studies of non-small cell lung cancer, 4 studies of stomach and/or gastroesophageal cancer, 3 studies each of melanoma and ovarian cancer, 2 studies each of breast cancer, transitional cell cancer, and small cell lung cancer, and 1 studies each of urothelial cancer, liver cancer, pancreatic cancer, colorectal cancer, endometrial cancer, multiple myeloma, and oropharyngeal cancer. Patients in the trial group received 15 regimens with pembrolizumab, 4 regimens with durvalumab, 3 regimens with nivolumab, 2 regimens with atezolizumab, and 2 regimens with avelumab. Tislelizumab, Sugemalimab, Cemiplimab, Serplulimab, Toripalimab were treated with 1 regimen each. All studies were registered and are available at ClinicalTrials.gov.

### 3.3 Risk of bias assessment

**Figure 2:**
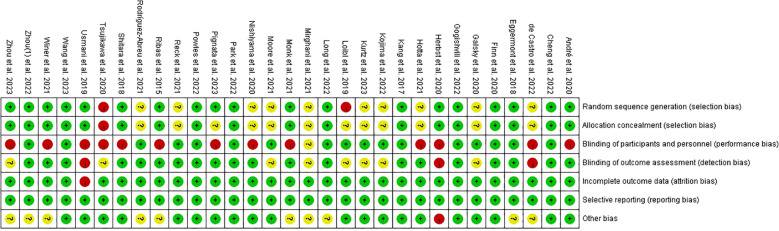
Risk of bias summary of included studies. Green, low risk of bias; yellow, unclear risk of bias; red, high risk of bias.

The Cochrane risk assessment tool was used to evaluate the quality of the included studies (**Figure 2**). All studies were RCTs, most of which used a central randomization system to achieve random sequence and allocation concealment; 19 were fully randomized; 18 reported explicit allocation concealment; and 20 were evaluated by a blinded Independent Central Review Committee, independent data Monitoring Committee, or independent center. To ensure the objectivity and accuracy of the research data, some research was funded by the pharmaceutical industry, which may have had some degree of bias. Of the 31 RCTs, 13 (42%) were open-label studies that did not blind participants and staff, and were classified as ambiguous for an unspecified risk of bias.

### 3.4 Incidence of IFI at any grade of ICPis vs. non-ICPis

**Figure 3:**
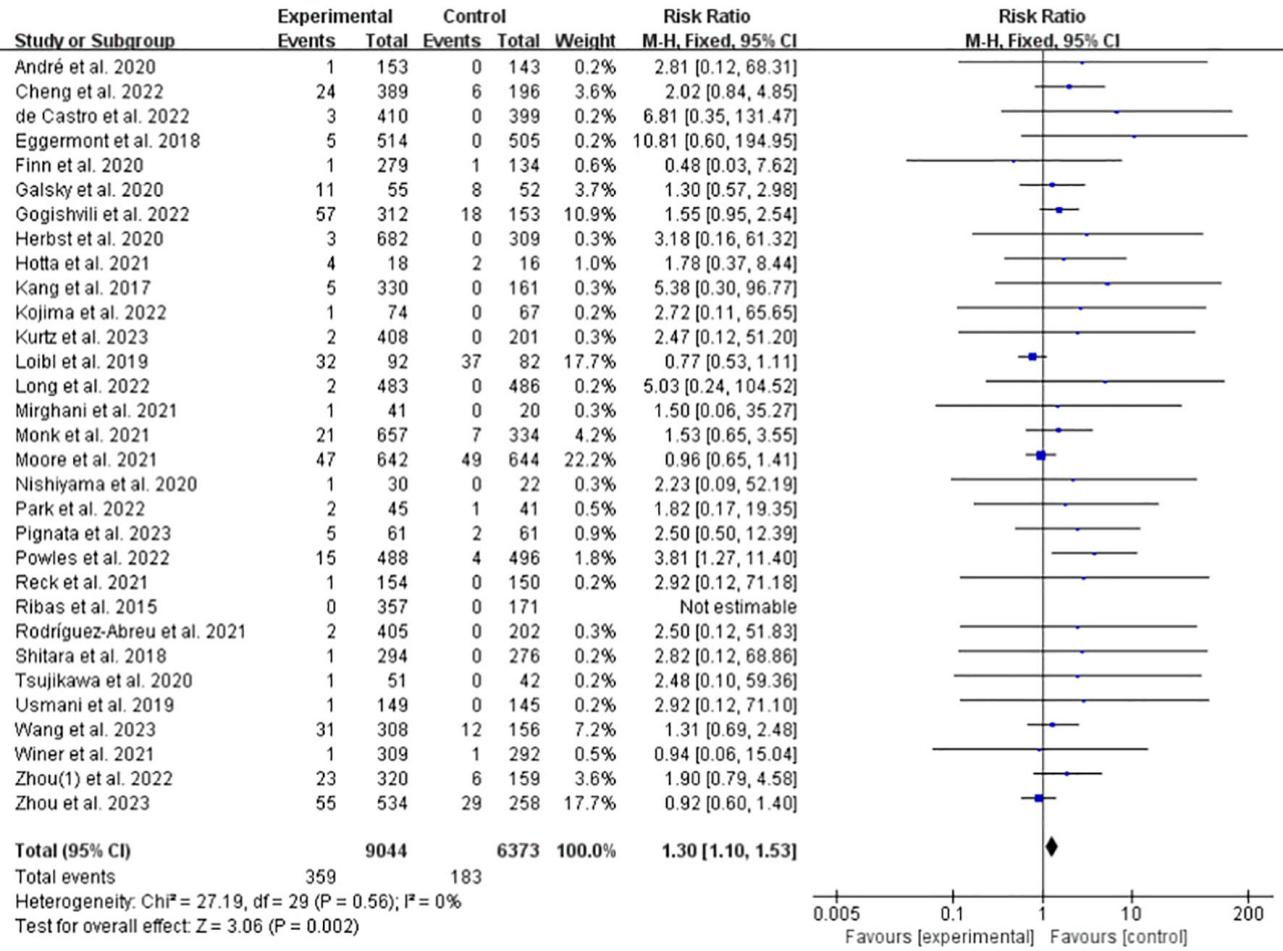
Incidence of IFI at any grade of ICPis experimental vs. non-ICPis control.

All the included studies clearly reported the number of patients with hyperglycemia, DM or DKA related to ICPis. Among them, 17 studies reported T1DM, 15 studies reported hyperglycemia, and 3 studies reported DKA; Among 15,417 patients, 542 had IFI-related events of all grades and 122 had serious adverse events (grade 3-5). Owing to the small number of T2DM events related to ICPis, it was clearly reported in only one study by Zhou et al. (1)., 2022 (25), the incidence rate was not statistically analyzed. Analysis of the data showed that the overall incidence of related IFI significantly increased in the ICPis group (RR = 1.30, 95%CI: 1.10-1.53, *P* = 0.002, ICPis: 359/9044 vs. non-ICPis: 183/6373) (**Figure 3**). The funnel plot is shown in **Figure S1** in **Supplementary Table 5**. The graph was symmetrical and no publication bias was observed. To evaluate the stability of the research results, we conducted a sensitivity analysis. After excluding each study item by item, the heterogeneity among the remaining studies did not change significantly, suggesting that the data and results of the 31 included RCT were stable and reliable (**Figure S2** in **Supplementary Table 5**). We used the Begg’s test to quantitatively detect the publication bias: Begg’s test (*P* = 0.8119) suggested no significant publication bias. Meanwhile, we observed the forest plot with *I*² = 0.0%, suggesting no heterogeneity. In summary, based on a pooled analysis of 31 RCTs, treatment with ICPis was associated with a statistically significant 30% increase in the relative risk of developing any-grade islet function impairment compared to non-ICPis therapies.

**Figure 4:**
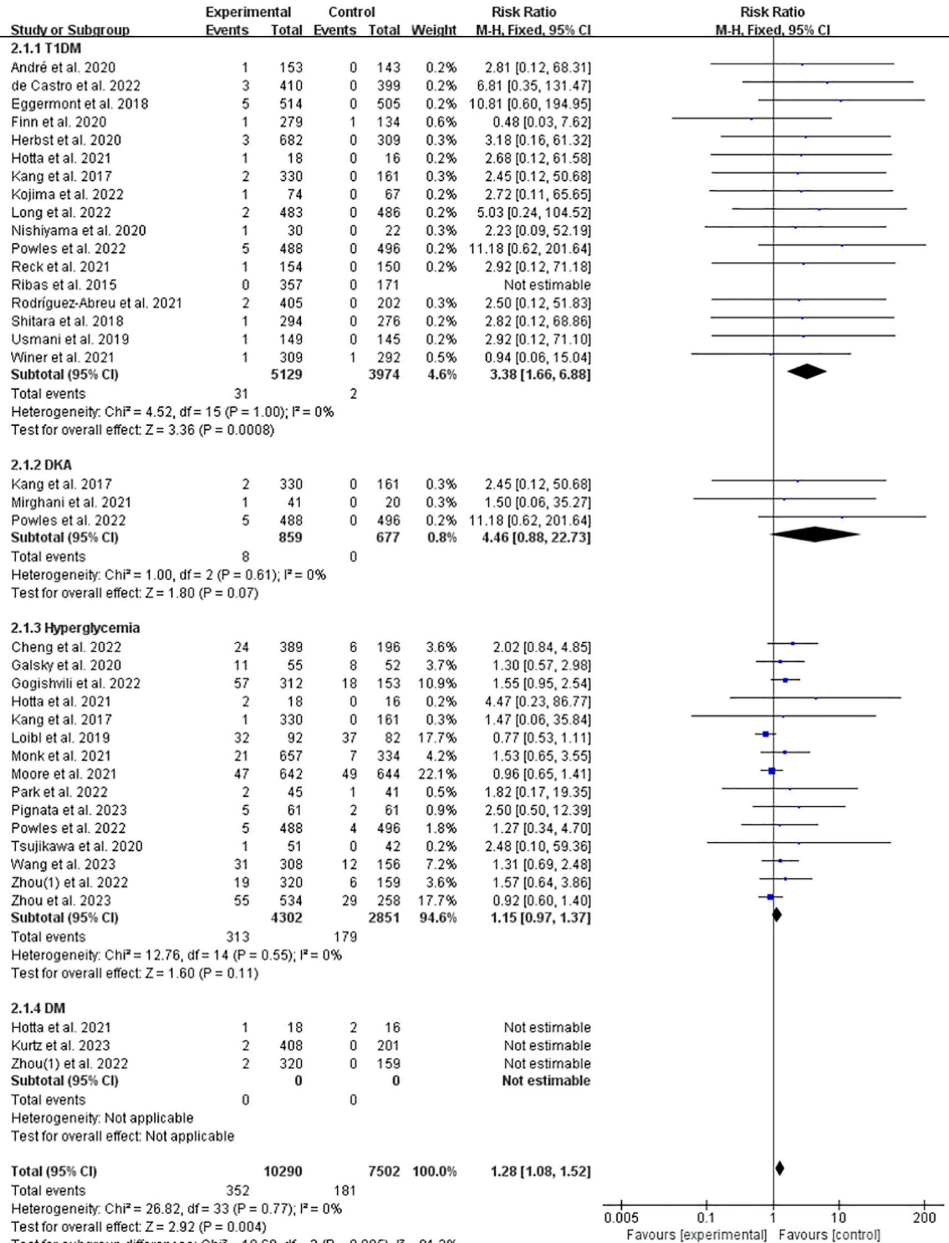
Incidence of T1DM/DKA/hyperglycemia/DM of ICPis experimental vs. non-ICPis control.

Subgroup analysis showed that the incidence of related T1DM significantly increased of ICPis group (RR = 3.38, 95%CI: 1.66-6.88, *P* = 0.0008, ICPis: 31/5129 vs. non-ICPis: 2/3974) (**Figure 4**). The funnel plot is shown in **Figure S3** in **Supplementary Table 5** Sensitivity analysis is shown in **Figure S4** in **Supplementary Table 5** Begg’s test *P* = 0.1275.

### 3.5 Incidence of IFI at grade 1–2 or grade 3–5 of ICPis vs. non-ICPis

**Figure 5:**
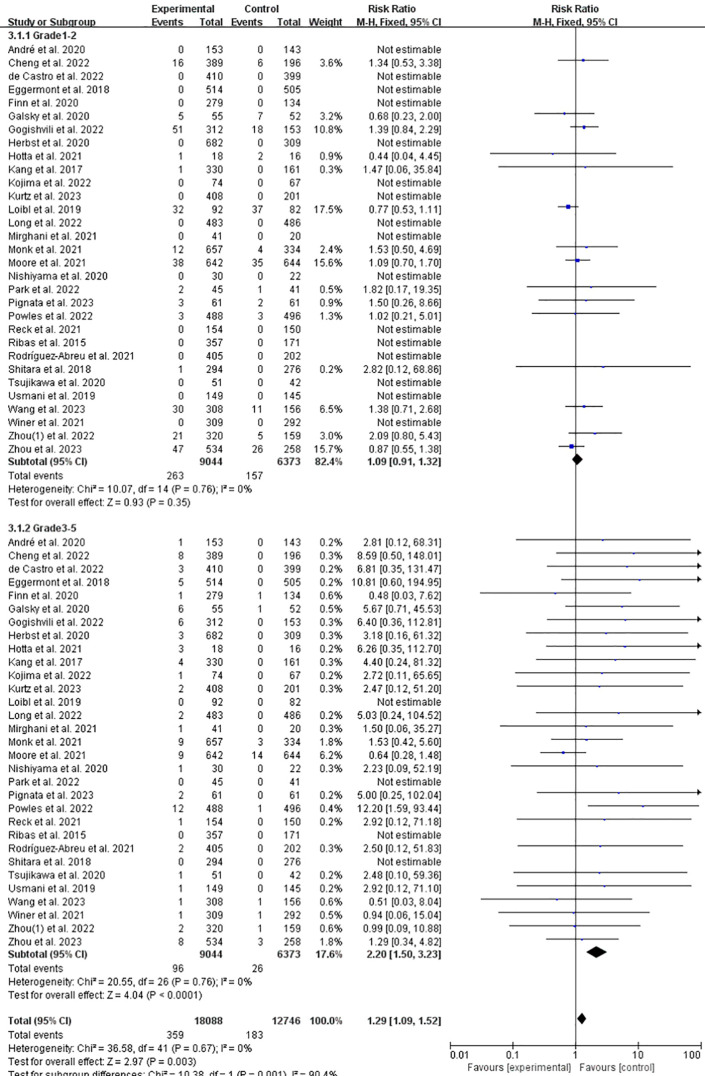
Incidence of IFI at grade 1–2 or grade 3–5 of ICPis experimental vs. non-ICPis control.

According to CTCAE, the adverse reactions related to ICPis were classified as follows: grade 1–2 were minor adverse events, and grade 3–5 were serious adverse events. Compared with the non-ICPis, the risk of grade 3–5 significantly increased (RR = 2.20, 95%CI: 1.50-3.23, *P* < 0.0001, ICPis: 96/9044 vs. non-ICPis: 26/6373) of ICPis. But there was no significant difference in the risk of grade 1-2 (RR = 1.09, 95%CI: 0.91-1.32, *P* = 0.35, ICPis: 263/9044 vs. non-ICPis: 157/6373) (**Figure 5**). In summary, while the risk of mild to moderate (grade 1-2) IFI was not significantly different between groups, ICPis therapy markedly increased the risk of severe, life-threatening, or fatal (grade 3-5) IFI events by more than twofold compared to control treatments. The funnel plot (**Figure S5** in **Supplementary Table 5**) showed no evident asymmetry, and Begg’s test (*P* = 0.1965) suggested a low likelihood of publication bias. Sensitivity analysis (**Figure S6** in **Supplementary Table 5**) confirmed the robustness of the primary finding for grade 3–5 events.

### 3.6 Incidence of IFI at any grade of PD-L1/PD-1 vs. non-ICPis

**Figure 6:**
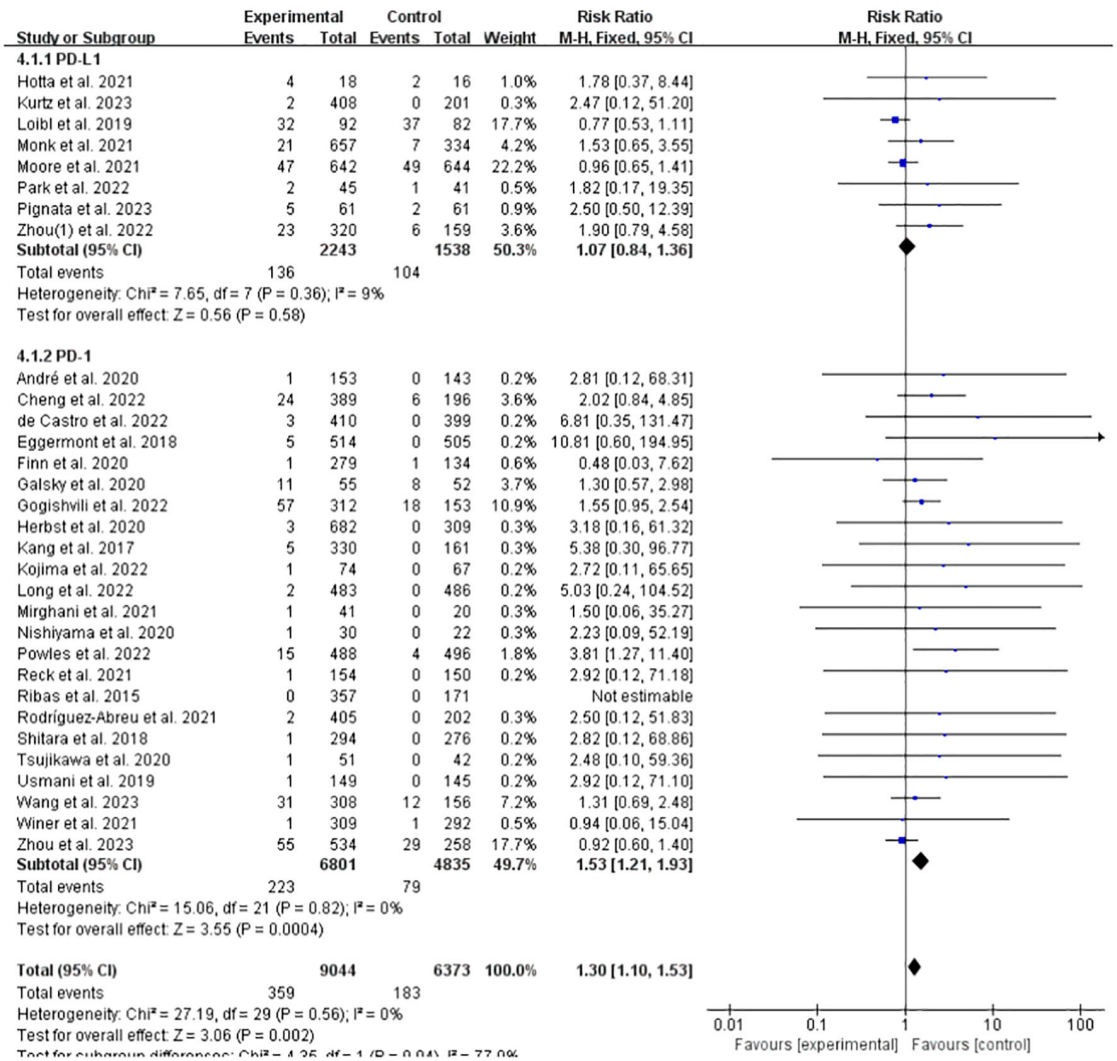
Incidence of IFI at any grade of PD-L1/PD-1 experimental vs. non-ICPis control.

Comparing the incidence of IFI related to CTLA-4, PD-1, and PD-L1 inhibitors after classifying and summarizing the relevant data, it was found that the incidence of IFI related to PD-L1 and PD-1 was 1.07 and 1.53 times that in the control group, respectively. Among them, the incidence of IFI in the PD-1 group increased significantly (RR = 1.53, 95%CI: 1.21-1.93, *P* = 0.0004, ICPis: 223/6801 vs. non-ICPis: 79/4835), and no significant difference was observed in the PD-L1 group (RR = 1.07, 95%CI: 0.84-1.36, *P* = 0.58, ICPis: 136/2243 vs. non-ICPis: 104/1538). Due to insufficient reports related to CTLA-4, no relevant statistical analyses were conducted (**Figure 6**). In summary, this analysis indicates that the significant overall increase in IFI risk associated with ICPis is primarily driven by PD-1 inhibitors, whereas PD-L1 inhibitors, based on the currently available RCT data, were not associated with a statistically significant elevation in risk. Assessment of publication bias for this subgroup analysis (Begg’s test *P* = 0.3156; funnel plot in **Figure S7** in **Supplementary Table 5**) did not indicate significant bias, and sensitivity analysis (**Figure S8** in **Supplementary Table 5**) supported the stability of the result for PD-1 inhibitors.

**Figure 7:**
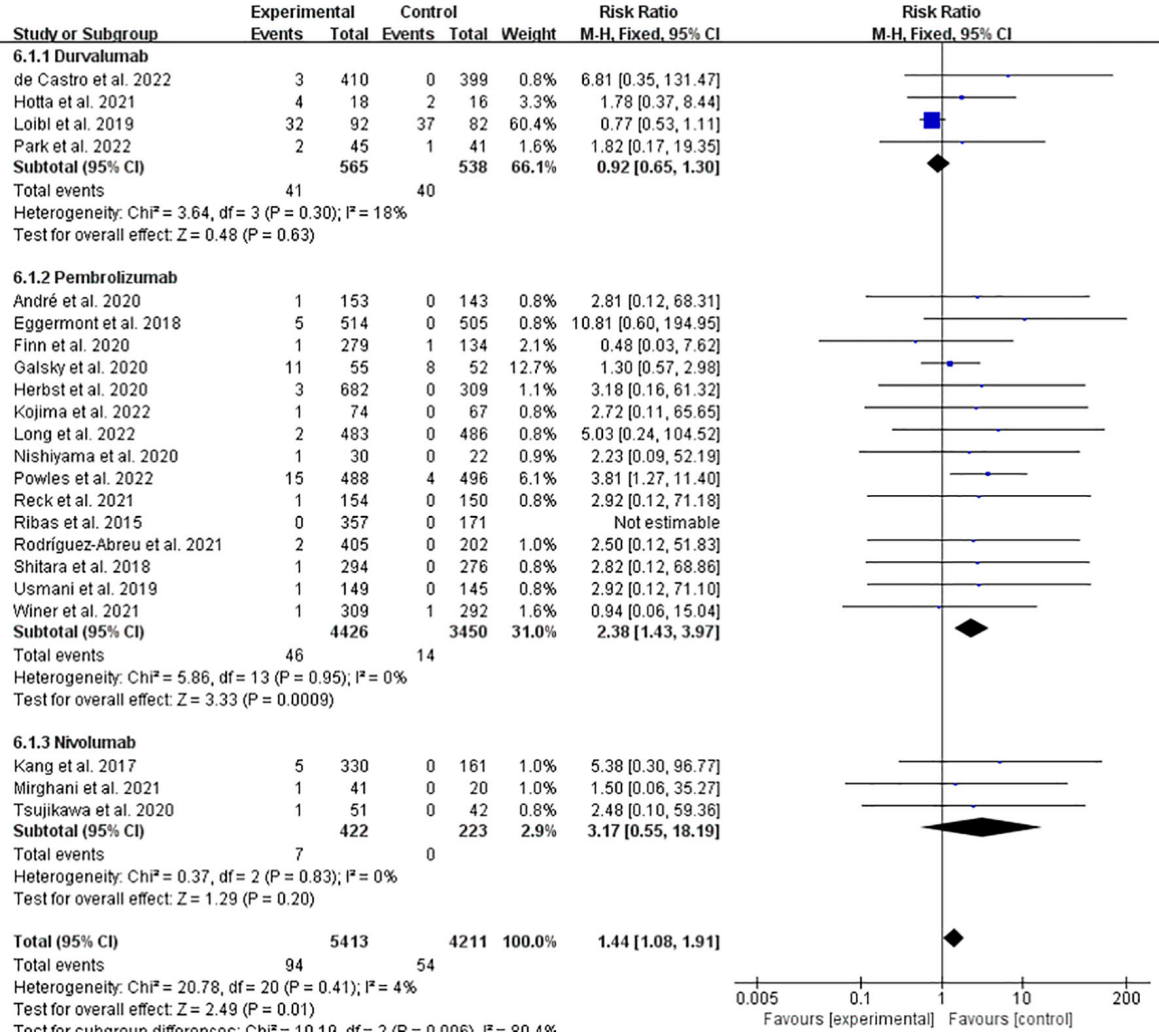
Incidence of IFI at any grade of Durvalumab/Pembrolizumab/Nivolumab experimental vs. non-ICPis control.

Meanwhile, we conducted subgroup analyses of the data for Durvalumab, Pembrolizumab and Nivolumab, which have been frequently reported. The results indicated that the incidence of IFI related to Pembrolizumab increased significantly (RR = 2.38, 95%CI: 1.43-3.97, *P* = 0.0009, ICPis: 46/4426 vs. non-ICPis: 14/3450), and no statistical difference was observed in the Durvalumab and Nivolumab groups (**Figure 7**). In summary, among the most frequently reported individual agents, pembrolizumab showed a pronounced and significant association with increased IFI risk. The point estimate for nivolumab was also above 1.0, suggesting a potential increase in risk, but it did not reach statistical significance in our analysis. The funnel plot is shown in **Figure S9** in **Supplementary Table 5** Sensitivity analysis is shown in **Figure S10** in **Supplementary Table 5** Begg’s test *P* = 0.4285.

### 3.7 Incidence of IFI at any grade of ICPis vs. non-ICPis in different tumors

**Figure 8:**
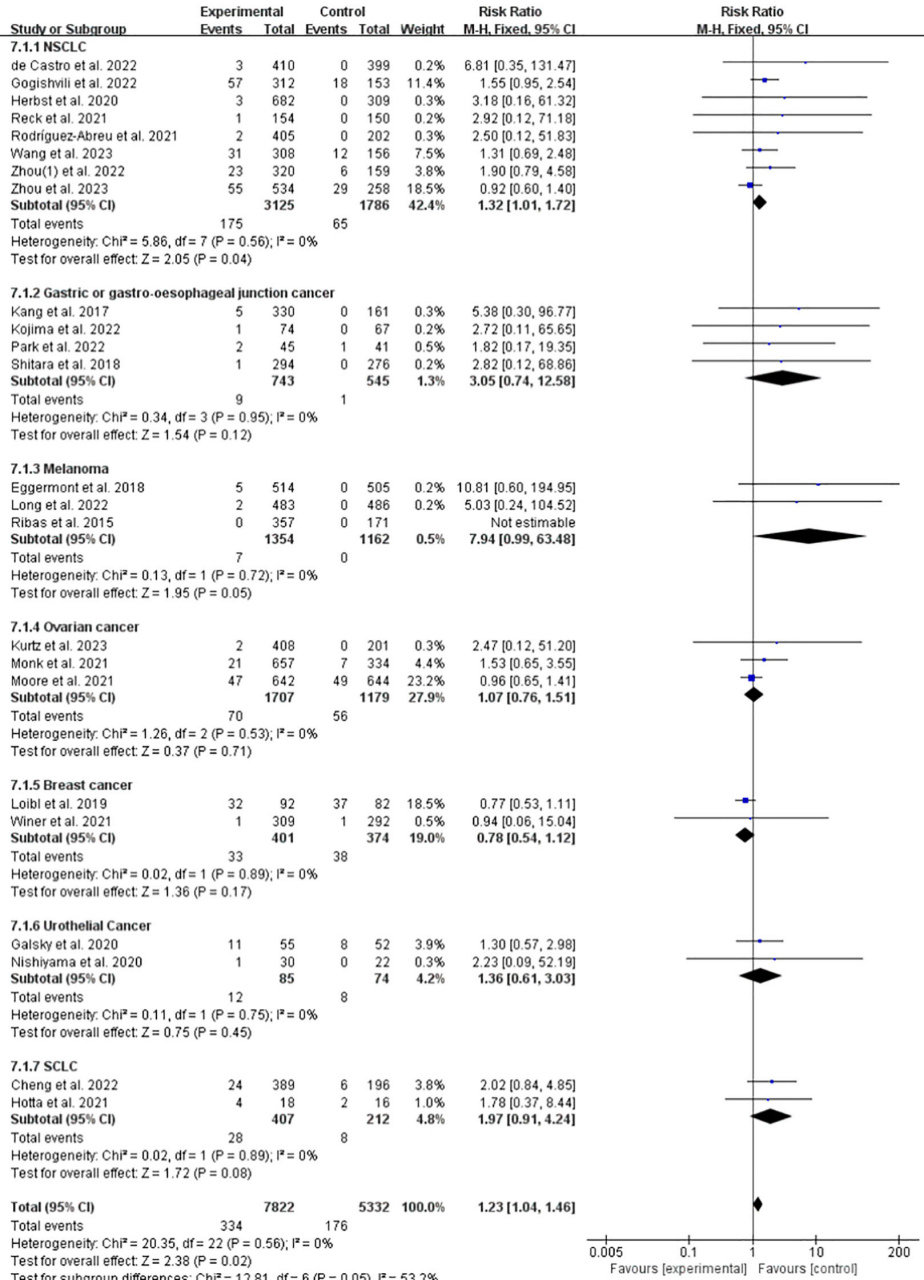
Incidence of IFI at any grade of ICPis experimental vs. non-ICPis control in different tumors.

To compare the incidence of IFI across different tumor types, we conducted a subgroup analysis based on tumor type. Compared with the control group, the incidence of IFI events after the use of ICPis in the NSCLC group significantly increased (RR = 1.32, 95%CI: 1.01-1.72, *P* = 0.04, ICPis: 175/3125 vs. non-ICPis: 65/1786), which was 1.32 times that in the control group (**Figure 8**). No statistically significant differences were observed among the other tumor types. The funnel plot is shown in **Figure S11** in **Supplementary Table 5** Sensitivity analysis is shown in **Figure S12** in **Supplementary Table 5** Begg’s test *P* = 0.7105.

### 3.8 Incidence of IFI at any grade of ICPis monotherapy/ICPis combined with chemotherapy vs. non-ICPis

**Figure 9:**
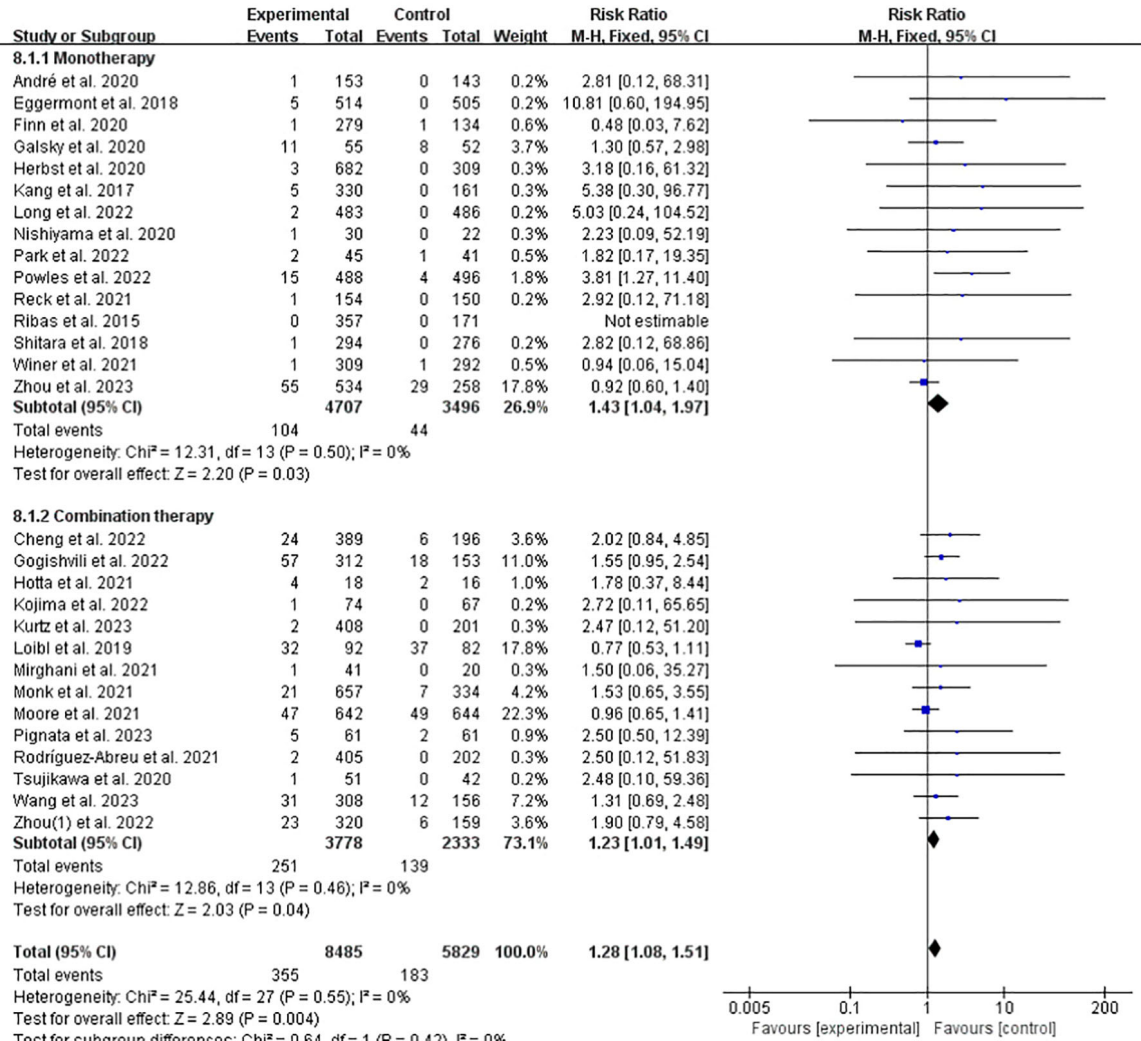
Incidence of IFI at any grade of ICPis monotherapy/ICPis combined with chemotherapy experimental vs. non-ICPis control.

**Figure 10:**
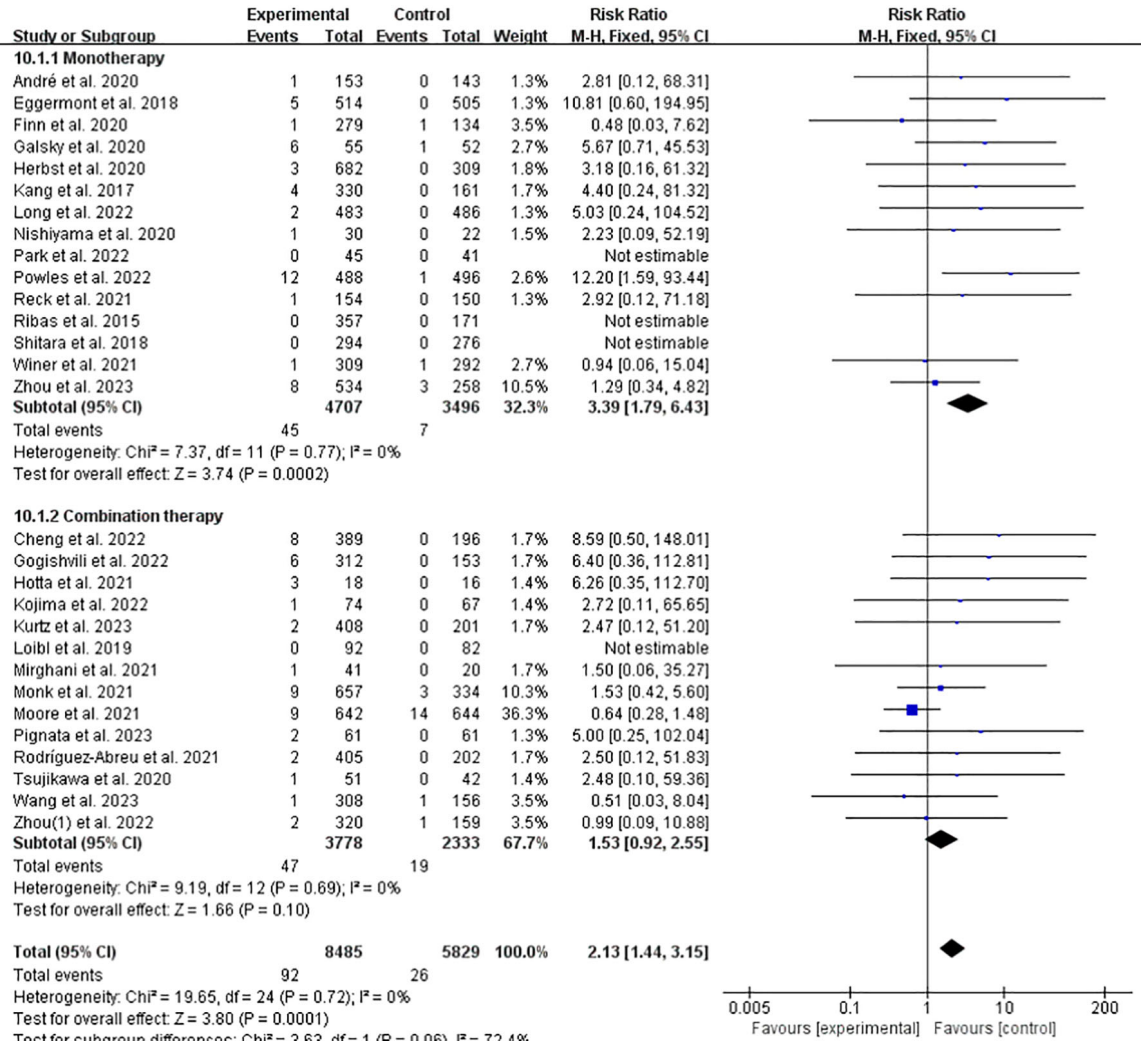
Incidence of IFI at grade 3–5 of ICPis monotherapy/ICPis combined with chemotherapy experimental vs. non-ICPis control.

Preclinical and clinical evidence indicates that chemotherapy regimens that can induce (or at least do not inhibit) anticancer immunity are particularly suitable in combination with ICPis. Therefore, we compared the combined treatment of chemotherapy and ICPis with ICPis monotherapy in terms of the incidence of related IFI. The risk of all-grade IFI was 1.23 times higher with ICPis plus chemotherapy and 1.43 times higher with ICPis monotherapy compared to non-ICPis controls (**Figure 9**). The funnel plot is shown in **Figure S13** in **Supplementary Table 5** The incidences of grade 3–5 related IFI were 1.53 and 3.39 times that of the control group respectively (**Figure 10**). Moreover, there was statistically significant difference in the incidence of grade 3–5 related IFI between the ICPis monotherapy experimental vs. non-ICPis control (RR = 3.39, 95%CI: 1.79-6.43, *P* = 0.0002, ICPis: 45/4707 vs. non-ICPis: 7/3496). However, there was no statistically significant difference between the ICPis combined chemotherapy experimental vs. non-ICPis control. In summary, when compared to their respective non-ICPis control arms, ICPis monotherapy conferred a significantly higher risk of severe IFI (RR = 3.39, 95%CI: 1.79–6.43, *P* = 0.0002, ICPis: 45/4707 vs. non-ICPis: 7/3496), whereas the risk associated with ICPis-chemotherapy combination therapy was lower and not statistically significant (RR = 1.53, 95%CI: 0.92–2.55, P = 0.10, ICPis: 47/3778 vs. non-ICPis: 19/2333). This suggests a differential risk profile between these two treatment modalities, with combination therapy potentially attenuating the severity of ICPis-related IFI. These findings were robust, as no significant publication bias was detected (Begg’s test *P* = 0.1133; **Figure S14** in **Supplementary Table 5**), and sensitivity analysis confirmed the stability of the results (**Figure S15** in **Supplementary Table 5**).

## 4 Discussion

Our study, encompassing 31 RCTs and over 15,000 patients, corroborates the established association between ICPis and IFI, while significantly extending the evidence base in several key dimensions. In contrast to prior meta-analyses that included mixed study designs, our RCT-exclusive approach minimizes bias and provides a higher level of evidence for causal inference. Compared to an earlier RCT-based meta-analysis, our work incorporates a substantially updated and larger dataset, reflecting the rapid evolution of ICPis therapy. The novel insights from our analysis include: (a) pinpointing PD-1 inhibitors (particularly pembrolizumab), but not PD-L1 inhibitors in our analysis, as the primary driver of increased IFI risk; (b) identifying non-small cell lung cancer (NSCLC) as a tumor type with a significantly elevated risk; (c) highlighting that the risk is particularly pronounced for grade 3–5 events and for type 1 diabetes mellitus (T1DM), underscoring its clinical severity; and (d) presenting the novel observation that ICPis combined with chemotherapy may be associated with a lower incidence of severe IFI compared to ICPis monotherapy, a finding requiring further mechanistic exploration. It is important to interpret the non-significant result for PD-L1 inhibitors with caution. While it may suggest a differential risk profile compared to PD-1 inhibitors, this finding could also be influenced by lower statistical power due to a smaller number of included RCTs and patients in this subgroup compared to the PD-1 inhibitor analysis. Similarly, the lack of significant association for individual agents like durvalumab and nivolumab, in contrast to pembrolizumab, may partly reflect their relatively smaller sample sizes in our dataset rather than an absence of risk.

Over the past decade, the development of ICPis has been a revolutionary breakthrough in the field of tumor treatment. It has ushered in a new era of antitumor therapy and has significantly improved the survival rate of patients with tumors. However, more than half of patients may experience immune-related adverse events. Among the relatively rare adverse reactions related to IFI, mild cases may present with symptoms, such as polydipsia, polyuria, and weight loss. In severe cases, symptoms of DKA, such as fatigue, nausea, vomiting, abdominal pain, dry skin, shortness of breath, breathing with a “rotten apple” smell, drowsiness, convulsions, or coma may occur (54). Unlike other endocrine adverse reactions, adverse reactions related to IFI are mostly acute onset and rapid progression, and the above hyperglycemic symptoms can occur within a short period. More than half of the patients are diagnosed with fulminant T1DM and are mainly treated with PD-1 and PD-L1 inhibitors. IFI related to CTLA-4 inhibitors is relatively rare, with only a few cases reported in the literature (12, 55, 56). Real-world research data indicate that IFI related to ICPis may occur within one day to a maximum of 1,771 days after the start of treatment, making it difficult to predict the occurrence time (4). Therefore, patients receiving ICPis treatment should undergo routine monitoring of blood glucose (GLU), glycated hemoglobin, and basic islet function (including C-peptide and pancreatic morphological changes) before initiating treatment (57). If ICPis-related IFI is considered, patients should be given dietary and lifestyle changes, as well as continuous GLU monitoring and insulin injection therapy. Notably, IFI is not a contraindication to continued treatment with PD-1 or PD-L1 inhibitors. Patients can receive subsequent ICPis treatment at the same time as starting insulin treatment for diabetes. However, in patients with severe diabetes, ICPis treatment may need to be delayed and continued after the condition improves (6). Unlike other endocrine system diseases, glucocorticoids are not recommended for patients with ICPis-related IFI. If patients need to be treated with high-dose glucocorticoids owing to other adverse reactions, continuous dynamic GLU monitoring should be strengthened to avoid deterioration of GLU levels caused by glucocorticoids (6).

Traditional chemotherapy preferentially kills rapidly proliferating malignant cells. Chemotherapy can also mediate immune stimulation by targeting tumors or immune cells and altering systemic physiological functions (58). Changes in the tumor immune microenvironment of patients with tumors can also affect the efficacy of chemotherapy. This provides a solid foundation for the development of new treatment regimens that combine traditional chemotherapy with ICPis-based immunotherapy (59). Our study showed that when non-ICPis treatment was used as the control group and ICPis treatment was used as the experimental group, the incidence of IFI in the experimental group increased significantly. However, when ICPis was used as an add-on therapy, the incidence decreased. Compared to their respective non-ICPis controls, the point estimate for IFI risk was lower with ICPis plus chemotherapy (RR = 1.23) than with ICPis monotherapy (RR = 1.43); a similar pattern was observed for grade 3–5 IFI (RR = 1.53 vs. 3.39). While these indirect comparisons suggest a trend toward lower IFI risk with combination therapy, this finding should be interpreted with caution and considered hypothesis-generating. Direct head-to-head comparisons in prospective trials are needed to confirm whether combining chemotherapy with ICPis genuinely attenuates the risk of severe IFI. The observed attenuation of severe IFI risk with ICPis-chemotherapy combination therapy is an intriguing finding that merits further investigation. Potential hypotheses include: (1) Immunomodulatory effects of chemotherapy: Certain agents may deplete or alter specific lymphocyte populations involved in autoimmune β-cell destruction. (2) Differences in treatment duration or immune activation context: Combination regimens might lead to a distinct temporal pattern of immune response. (3) Confounding by patient selection: Patients eligible for intensive combination therapy may have different baseline characteristics. However, this is an indirect comparison derived from subgroup analyses, and the finding should be considered hypothesis-generating (60). Future preclinical studies and prospective clinical trials with careful biomarker collection are needed to elucidate any potential protective mechanism. ICPis combined with chemotherapy have been tested in a variety of solid tumors, achieving a synergistic effect and overcoming drug resistance in immunotherapy (59, 61). Increasing evidence supports the clinical value of combining appropriate doses of chemotherapeutic with ICPis. In March 2019 and March 2020, the FDA approved the combination of Atezolizumab and Durvalumab combined with chemotherapy as a first-line treatment for patients with extensive-stage small cell lung cancer, which can significantly improve the overall survival (62).

This meta-analysis showed that the risk of grade 3–5 IFI and T1DM significantly increased after ICPis treatment. However, this finding does not indicate that ICPis treatment is significantly associated with an increased risk of DKA or hyperglycemia. This might be due to the small number of studies included. However, the incidence of ICPis-related DKA was 4.46 times that of the control group. Among IFI cases related to ICPis, the risk of life-threatening adverse events is relatively high and requires focused clinical attention. However, the pathogenesis of ICPis-related diabetes remains unclear. Animal models indicate that inhibition or deficiency of PD-1 or PD-L1 can lead to rapid development of diabetes in mice (63–65). *In vivo* studies have shown that PD-L1 is not only widely expressed in lymphoid tissue, but also expressed in pancreatic cells (66), Therefore, blocking the interaction between PD-1 and PD-L1 may stimulate the proliferation and activation of T cells, thereby leading to cell destruction, which provides the possibility for PD-1 inhibitors to induce T1DM (4). Furthermore, compared with the healthy control group or patients with T2DM, the expression of PD-1 in T cells of patients with T1DM is lower (67, 68), Peripheral CD4^+^ and regulatory T cells showed decreased PD-1 expression. The co-occurrence of ICPis therapy and T1DM may reflect underlying PD-1 pathway dysregulation. Case reports have indicated that the presence of autoantibodies before ICPis-based treatment may pose a risk of diabetes, especially during treatment with PD-1 and PD-L1 inhibitors (69–71). Clotman et al. (72) further support a mechanism based on autoimmunity. They outlined the reported cases and demonstrated that approximately half of ICPis-related T1DM test cases had detectable diabetes-related autoantibodies. Further studies have found that 76% of patients with diabetes and ICPis carry the T1DM susceptibility gene HLA-DR4 (12, 73), which has led researchers to believe that genetic factors may be a possible mechanism for patients with HLA genotypes to be prone to diabetes. These studies have revealed potential mechanisms underlying ICPis-related IFI, including the immune and genetic factors associated with diabetes. Therefore, managing ICPis-related IFI necessitates a proactive, multidisciplinary team approach, involving close collaboration between oncologists, immunologists, endocrinologists, diabetes educators, and pharmacists. Institutional protocols should be established for baseline risk assessment (e.g., glucose, HbA1c, optional pancreatic autoantibodies) and regular monitoring during treatment, especially in high-risk groups identified herein.

The limitations of this study are as follows. (1) Many other factors, such as demographic characteristics (age, sex, or race) and previous treatments received, may also interfere with the meta-analysis. (2) 22/31 studies involved PD-1 inhibitors, 8/31 involved PD-L1 inhibitors, and 1/31 involved CTLA-4 inhibitors. Owing to the imbalance of the datasets, it is challenging to compare or summarize the related IFI of different ICPis. (3) In studies comparing the relationships between the incidence of different types of tumors, ICPis, and different types of IFI, the number of included studies and the sample size were relatively small. (4) Potential errors in data extraction or study selection cannot be ruled out even in cases of missing or repetitive research. (5) There is a lack of detailed individual clinical data, such as gender, HLA genotypes prone to diabetes, the presence of autoantibodies, and islet function in patients receiving ICPis treatment. Therefore, it is impossible to comprehensively assess the potential risk factors associated with the high risk of IFI.

## 5 Conclusion

This updated RCT-exclusive meta-analysis confirms that ICPis—particularly PD-1 inhibitors such as pembrolizumab—significantly increase the risk of IFI, with markedly elevated risks for T1DM and grade 3–5 events. Patients with NSCLC are identified as a particularly vulnerable population. Although combination with chemotherapy showed lower risk estimates in indirect comparisons, this finding requires prospective validation. These results underscore the urgent need for proactive, multidisciplinary monitoring strategies tailored to high-risk patients receiving ICPis. However, the certainty of evidence was moderate to very low for several outcomes (**Supplementary Table 4**). Future large-scale, well-designed RCTs are warranted to elucidate the underlying mechanisms and to optimize the safety profile of ICPis.

## Data Availability

The original contributions presented in the study are included in the article/**Supplementary Material**. Further inquiries can be directed to the corresponding authors.

## Glossary

ICPis: immune checkpoint inhibitors
RCT: randomized controlled trial
DKA: diabetic ketoacidosis
CTLA-4: cytotoxic T lymphocyte-associated antigen-4 inhibitors
PD-L1: programmed cell death-ligand 1
PD-1: programmed death-1 inhibitors
T2DM: type 2 diabetes mellitus
T1DM: type 1 diabetes mellitus
DM: diabetes mellitus
CI: confidence interval
GLU: blood glucose
