# Supplementary material for: Islet function impairment outcomes of immune checkpoint inhibitors in cancer patients: a systematic review and meta-analysis
Source: Front Immunol. 2026 Mar 19;17:1669492. doi: 10.3389/fimmu.2026.1669492 (PMC13044012; doi:10.3389/fimmu.2026.1669492)
Supplement: Supplementary file 2 [file Table2.docx]

**Table2.**

**Draft of search strategy**

**Search strategy in PubMed**

PubMed search:

 ((Diabetes) OR ("Endocrine System Diseases"[Mesh])) AND (("Immune Checkpoint Inhibitors"[Mesh] OR "Immune Checkpoint Inhibitors" [Pharmacological Action] OR "Immune Checkpoint Proteins"[Mesh]) OR (atezolizumab OR avelumab OR tremllimumab OR nivolumab OR pembrolizumab OR toripalimab OR sintilimab OR tislelizumab OR cemiplimab OR durvalumab OR ipilimumab OR tremelimumab OR lisavanbulin OR sotorasib OR spartalizumab OR relatlimab OR PHI-101 OR quemliclustat OR Abatacept OR Cadonilimab OR "Polatuzumab Vedotin"OR "Trastuzumab Deruxtecan" OR Camrelizumab OR ICPis OR "Programmed Cell Death 1Receptor" OR "PD-1 Receptor" OR "CD279 Antigen" OR "CTLA-4" OR "Anti-cytotoxic T lymphocyte associated antigen-4" OR "anti-programmed cell death-1" OR "anti-programmed cell death ligand-1" OR Serplulimab OR Zimberelimab OR Penpulimab OR Envafolimab OR Sugemalimab OR "Immune checkpoint inhibitor" OR anti–PD-L1 OR PD-1 OR PD-L1 OR "Programmed death-1" OR "Programmed death ligand-1" OR MDX-010 OR MK-3475 OR MPDL3280A OR BMS-963558)) Filters: Randomized Controlled Trial. (n=84)

**Search strategy in cochrane library**

cochrane library search:

(atezolizumab OR avelumab OR tremllimumab OR nivolumab OR pembrolizumab OR toripalimab OR sintilimab OR tislelizumab OR cemiplimab OR durvalumab OR ipilimumab OR tremelimumab OR lisavanbulin OR sotorasib OR spartalizumab OR relatlimab OR PHI-101 OR quemliclustat OR Abatacept OR Cadonilimab OR Polatuzumab VedotinOR Trastuzumab Deruxtecan OR Camrelizumab OR ICPis OR Programmed Cell Death 1Receptor OR PD-1 Receptor OR CD279 Antigen OR CTLA-4 OR Anti-cytotoxic T lymphocyte associated antigen-4 OR anti-programmed cell death-1 OR anti-programmed cell death ligand-1 OR Serplulimab OR Zimberelimab OR Penpulimab OR Envafolimab OR Sugemalimab OR Immune checkpoint inhibitor OR anti–PD-L1 OR PD-1 OR PD-L1 OR Programmed death-1 OR Programmed death ligand-1 OR MDX-010 OR MK-3475 OR MPDL3280A OR BMS-963558) AND Diabete:155条；（Embase109,Pubmed72,ICTRR15,clinical trail15）. (n=155)

**Search strategy in Clinical Trail**

Clinical Trail:

(Checkpoint Inhibitors, Immune OR Immune Checkpoint Blockers OR Checkpoint Blockers, Immune OR Immune Checkpoint Inhibitor OR Checkpoint Inhibitor, Immune OR CTLA-4 Inhibitors OR CTLA 4 Inhibitors OR Cytotoxic T-Lymphocyte-Associated Protein 4 Inhibitors OR Cytotoxic T Lymphocyte Associated Protein 4 Inhibitors OR Cytotoxic T-Lymphocyte-Associated Protein 4 Inhibitor OR Cytotoxic T Lymphocyte Associated Protein 4 Inhibitor OR CTLA-4 Inhibitor OR CTLA 4 Inhibitor OR PD-1 Inhibitors OR PD 1 Inhibitors OR Programmed Cell Death Protein 1 Inhibitor OR Programmed Cell Death Protein 1 Inhibitors OR PD-1 Inhibitor OR Inhibitor, PD-1 OR PD 1 Inhibitor OR Immune Checkpoint Blockade OR Checkpoint Blockade, Immune OR Immune Checkpoint Inhibition OR Checkpoint Inhibition, Immune OR PD-L1 Inhibitors OR PD L1 Inhibitors OR Programmed Death-Ligand 1 Inhibitors OR Programmed Death Ligand 1 Inhibitors OR PD-L1 Inhibitor OR PD L1 Inhibitor OR PD-1-PD-L1 Blockade OR Blockade, PD-1-PD-L1 OR PD 1 PD L1 Blockade OR Immune Checkpoint Molecule OR Checkpoint Molecule, Immune OR Molecule, Immune Checkpoint OR Immune Checkpoint Molecules OR Immune Checkpoint Protein OR Checkpoint Protein, Immune OR Protein, Immune Checkpoint OR Stimulatory Checkpoint Molecules OR Stimulatory Checkpoint Molecule OR Checkpoint Molecule, Stimulatory OR Inhibitory Checkpoint Molecules OR Inhibitory Checkpoint Molecule OR Checkpoint Molecule, Inhibitory OR atezolizumab OR avelumab OR tremllimumab OR nivolumab OR pembrolizumab OR toripalimab OR sintilimab OR tislelizumab OR cemiplimab OR durvalumab OR ipilimumab OR tremelimumab OR lisavanbulin OR sotorasib OR spartalizumab OR relatlimab OR PHI-101 OR quemliclustat OR Abatacept OR Cadonilimab OR “Polatuzumab Vedotin”OR “Trastuzumab Deruxtecan” OR Camrelizumab OR ICPis OR “Programmed Cell Death 1Receptor” OR “PD-1 Receptor” OR “CD279 Antigen” OR “CTLA-4” OR “Anti-cytotoxic T lymphocyte associated antigen-4” OR “anti-programmed cell death-1” OR “anti-programmed cell death ligand-1” OR Serplulimab OR Zimberelimab OR Penpulimab OR Envafolimab OR Sugemalimab OR “Immune checkpoint inhibitor” OR anti–PD-L1 OR PD-1 OR PD-L1 OR “Programmed death-1” OR “Programmed death ligand-1” OR MDX-010 OR MK-3475 OR MPDL3280A OR BMS-963558 OR Dostarlimab) AND (Type 1 Diabetes OR Diabetes, Type 1 OR Diabetes Mellitus, Insulin-Dependent OR Diabetes Mellitus, Insulin Dependent OR Insulin-Dependent Diabetes Mellitus OR Diabetes Mellitus, Juvenile-Onset OR Diabetes Mellitus, Juvenile Onset OR Juvenile-Onset Diabetes Mellitus OR IDDM OR Diabetes Mellitus, Type I OR Diabetes Mellitus, Sudden-Onset OR Diabetes Mellitus, Sudden Onset OR Sudden-Onset Diabetes Mellitus OR Type 1 Diabetes Mellitus OR Diabetes Mellitus, Insulin-Dependent, 1 OR Insulin-Dependent Diabetes Mellitus 1 OR Insulin Dependent Diabetes Mellitus 1 OR Juvenile-Onset Diabetes OR Diabetes, Juvenile-Onset OR Juvenile Onset Diabetes OR Diabetes, Autoimmune OR Autoimmune Diabetes OR Diabetes Mellitus, Brittle OR Brittle Diabetes Mellitus OR Diabetes Mellitus, Ketosis-Prone OR Diabetes Mellitus, Ketosis Prone OR Ketosis-Prone Diabetes Mellitus) （n=129）

**Search strategy in Embase**

**Embase search:**

#1 'checkpoint inhibitors, immune' OR 'immune checkpoint blockers' OR 'checkpoint blockers, immune' OR 'checkpoint inhibitor, immune' OR 'ctla-4 inhibitors' OR 'ctla 4 inhibitors' OR 'cytotoxic t-lymphocyte-associated protein 4 inhibitors' OR 'cytotoxic t lymphocyte associated protein 4 inhibitors' OR 'cytotoxic t-lymphocyte-associated protein 4 inhibitor' OR 'cytotoxic t lymphocyte associated protein 4 inhibitor' OR 'ctla-4 inhibitor' OR 'ctla 4 inhibitor' OR 'pd-1 inhibitors' OR 'pd 1 inhibitors' OR 'programmed cell death protein 1 inhibitor'/exp OR 'programmed cell death protein 1 inhibitors' OR 'pd-1 inhibitor' OR 'inhibitor, pd-1' OR 'pd 1 inhibitor'/exp OR 'immune checkpoint blockade'/exp OR 'checkpoint blockade, immune' OR 'immune checkpoint inhibition'/exp OR 'checkpoint inhibition, immune' OR 'pd-l1 inhibitors' OR 'pd l1 inhibitors' OR 'programmed death-ligand 1 inhibitors' OR 'programmed death ligand 1 inhibitors' OR 'pd-l1 inhibitor' OR 'pd l1 inhibitor'/exp OR 'pd-1-pd-l1 blockade' OR 'blockade, pd-1-pd-l1' OR 'pd 1 pd l1 blockade' OR 'immune checkpoint molecule' OR 'checkpoint molecule, immune' OR 'molecule, immune checkpoint' OR 'immune checkpoint molecules' OR 'immune checkpoint protein'/exp OR 'checkpoint protein, immune' OR 'protein, immune checkpoint' OR 'stimulatory checkpoint molecules' OR 'stimulatory checkpoint molecule' OR 'checkpoint molecule, stimulatory' OR 'inhibitory checkpoint molecules' OR 'inhibitory checkpoint molecule' OR 'checkpoint molecule, inhibitory' OR 'atezolizumab'/exp OR 'avelumab'/exp OR tremllimumab OR 'nivolumab'/exp OR 'pembrolizumab'/exp OR 'toripalimab'/exp OR 'sintilimab'/exp OR 'tislelizumab'/exp OR 'cemiplimab'/exp OR 'durvalumab'/exp OR 'ipilimumab'/exp OR 'tremelimumab'/exp OR 'lisavanbulin'/exp OR 'sotorasib'/exp OR 'spartalizumab'/exp OR 'relatlimab'/exp OR 'phi 101' OR 'quemliclustat'/exp OR 'abatacept'/exp OR 'cadonilimab'/exp OR 'trastuzumab deruxtecan'/exp OR 'camrelizumab'/exp OR icpis OR 'programmed cell death 1receptor' OR 'pd-1 receptor' OR 'cd279 antigen'/exp OR 'ctla-4'/exp OR 'anti-cytotoxic t lymphocyte associated antigen-4' OR 'anti-programmed cell death-1' OR 'anti-programmed cell death ligand-1' OR 'serplulimab'/exp OR 'zimberelimab'/exp OR 'penpulimab'/exp OR 'envafolimab'/exp OR 'sugemalimab'/exp OR 'immune checkpoint inhibitor'/exp OR 'anti–pd l1' OR 'pd 1'/exp OR 'pd l1' OR 'programmed death-1' OR 'programmed death ligand-1'/exp OR 'mdx 010'/exp OR 'mk 3475'/exp OR 'mpdl3280a'/exp OR 'bms 963558'

#2 't1dm' OR 'type 1 diabetes' OR 'diabetes, type 1' OR 'diabetes mellitus, insulin-dependent' OR 'diabetes mellitus, insulin dependent' OR 'insulin-dependent diabetes mellitus' OR 'diabetes mellitus, juvenile-onset' OR 'diabetes mellitus, juvenile onset' OR 'juvenile-onset diabetes mellitus' OR iddm OR 'diabetes mellitus, type i' OR 'diabetes mellitus, sudden-onset' OR 'diabetes mellitus, sudden onset' OR 'sudden-onset diabetes mellitus' OR 'type 1 diabetes mellitus' OR 'diabetes mellitus, insulin-dependent, 1' OR 'insulin-dependent diabetes mellitus 1' OR 'insulin dependent diabetes mellitus 1' OR 'juvenile-onset diabetes' OR 'diabetes, juvenile-onset' OR 'juvenile onset diabetes' OR 'diabetes, autoimmune' OR 'autoimmune diabetes' OR 'diabetes mellitus, brittle' OR 'brittle diabetes mellitus' OR 'diabetes mellitus, ketosis-prone' OR 'diabetes mellitus, ketosis prone' OR 'ketosis-prone diabetes mellitus' OR diabete OR hyperglycemia OR 'diabetic ketoacidosis'

#1 AND #2 AND [randomized controlled trial]/lim AND [humans]/lim（Embase and MEDLINE: 146+Embase: 54）(n=200)

**Search strategy in Chinese Wanfang**

**Wanfang:**

[Mesh]: (Immune checkpoint inhibitors) and [Mesh]: (Diabetes). (n=152)

**Search strategy in CNKI**

**CNKI:**

(Subject: Immune checkpoint inhibitors (Exact match)) AND (Subject: Diabetes (Exact match)). (n=65 results)

**Search strategy in China VIP**

**China VIP:**

Subject:(Immune checkpoint inhibitors) and Subject: (Diabetes). (n=113)

**Search strategy in SinoMed**

**SinoMed:**

(((“Immune checkpoint inhibitors”[all fields] OR “Immune Checkpoint Inhibitors”[all fields] OR “Programmed Cell Death Protein 1 inhibitors”[all fields] OR “PD-1 inhibitors”[all fields] OR “PD-1-PD-L1 blockade”[all fields] OR “Immune Checkpoint Blockers”[all fields] OR “Immune Checkpoint Blockade”[all fields] OR “Immune Checkpoint Inhibition”[all fields] OR “PD-L1 inhibitors”[all fields] OR “Programmed Death Ligand 1 inhibitors”[all fields] OR “CTLA-4 inhibitors”[all fields] OR “Cytotoxic T Lymphocyte Associated Protein 4 inhibitors”[all fields] OR “Immune Checkpoint Inhibitors”[keywords]) AND (“Diabetes”[all fields] OR “Diabetes Mellitus”[all fields] OR “Diabetes”[keywords])) AND “Clinical Trials”[document type]) AND -2024[date]. (n=4)
