# Supplementary material for: Islet function impairment outcomes of immune checkpoint inhibitors in cancer patients: a systematic review and meta-analysis
Source: Front Immunol. 2026 Mar 19;17:1669492. doi: 10.3389/fimmu.2026.1669492 (PMC13044012; doi:10.3389/fimmu.2026.1669492)
Supplement: Supplementary file 3 [file Table3.docx]

| Table3. Characteristics of the total study population | | | | | | | | | | | | |
| --- | --- | --- | --- | --- | --- | --- | --- | --- | --- | --- | --- | --- |
| **First author（year）** | **Country** | **Clinical trial identification** | **Phase** | **Patients No. (Male %)** | **Age, years, median (Experimental/control)** | **Tumor** | **ICPis** | **Experimental group** | **No. of Experimental group** | **control group** | **No. Of control group** | **Primary Outcome Measures** |
| André, 2020 | 23 countries | NCT02563002 | Ⅲ | 307（49.84） | 63/62.5 | Colorectal Cancer | PD-1 | Pembrolizumab | 153 | Chemotherapy | 143 | T1DM |
| Cheng, 2022 | 6 countries | NCT04063163 | Ⅲ | 585(82.22) | 63/62 | Small Cell Lung Cancer | PD-1 | Serplulimab | 389 | Placebo+carboplatin + etoposide | 196 | Hyperglycemia |
| de Castro, 2022 | 29 countries | NCT02542293 | Ⅲ | 823(73.1) | 64 | NSCLC | PD-L1+CTLA-4 | Durvalumab + Tremelimumab | 410 | Chemotherapy | 399 | T1DM |
| Eggermont, 2018 | 23 countries | NCT02362594 | Ⅲ | 1019（62） | 54/54 | Melanoma | PD-1 | Pembrolizumab | 514 | placebo | 505 | T1DM |
| Finn, 2020 | 27 countries | NCT02702401 | Ⅲ | 413（81.84） | 67/65 | Hepatocellular Carcinoma | PD-1 | Pembrolizumab | 279 | Placebo | 134 | T1DM |
| Galsky, 2020 | USA | NCT02500121 | II | 107(75.7) | 68/65 | Urothelial Cancer | PD-1 | Pembrolizumab | 55 | Placebo | 52 | Hyperglycaemia |
| Gogishvili, 2022 | 10 countries | NCT03409614 | Ⅲ | 466（84） | 63 | NSCLC | PD-1 | Cemiplimab | 312 | Placebo+Chemotherapy | 153 | Hyperglycemia |
| Herbst, 2020 | 24 countries | NCT01905657 | II/Ⅲ | 724(87.57) | 63/63/62 | NSCLC | PD-1 | Pembrolizumab | 339 343 | Docetaxel | 309 | T1DM |
| Hotta, 2021 | Japan | NCT03043872 | Ⅲ | 34(82.35) | 67.5/69.5 | Small Cell Lung Cancer | PD-L1 | Durvalumab | 18 | etoposide + cisplatin or carboplatin | 16 | DM；Hyperglycemia；T1DM |
| Kang, 2017 | Japan,  South Korea | NCT02267343 | Ⅲ | 493(70.59) | 62/61 | Gastric or gastro-oesophageal junction cancer | PD-1 | Nivolumab | 330 | Placebo | 161 | Hyperglycaemia;DKA; T1DM |
| Kojima, 2022 | Japan | NCT03189719 | Ⅲ | 141（87.94） | 68/68 | Esophageal cancer | PD-1 | Pembrolizumab | 74 | Placebo+chemotherapy | 67 | T1DM |
| Kurtz, 2023 | 74 sites in Europe and Israel | NCT02891824 | Ⅲ | 609 | 63/64 | Ovarian cancer | PD-L1 | Atezolizumab | 408 | placebo+ bevacizumab + chemotherapy | 201 | DM |
| Loibl, 2019 | German | NCT02685059 | II | 174 | 49.5 | Breast cancer | PD-L1 | Durvalumab | 92 | placebo+nab-paclitaxel + epirubicin/cyclophosphamide | 82 | Hyperglycemia |
| Long, 2022 | 16 countries | NCT03553836 | Ⅲ | 976(60.35) | 60/61 | Melanoma | PD-1 | Pembrolizumab | 483 | Placebo | 486 | T1DM |
| Mirghani, 2021 | France | NCT03838263 | II | 61(77) | 60 | Oropharynx cancer | PD-1 | Nivolumab | 41 | cisplatin-based chemoradiation | 20 | DKA |
| Monk, 2021 | 25 countries | NCT02718417 | Ⅲ | 998 | 59/60/57 | Ovarian cancer | PD-L1 | Avelumab | 329 / 328 | chemotherapy | 334 | Hyperglycemia |
| Moore, 2021 | North and South America, Europe, Asia, and Australia | NCT03038100 | Ⅲ | 1301 | 60/59 | Ovarian cancer | PD-L1 | Atezolizumab | 642 | placebo + paclitaxel + carboplatin + bevacizumab | 644 | Hyperglycemia |
| Nishiyama, 2020 | Japan | NCT02256436 | Ⅲ | 52(76.92) | 72/70.5 | Urothelial cancer | PD-1 | Pembrolizumab | 30 | Chemotherapy | 22 | T1DM |
| Park, 2022 | Korea/USA | NCT02520453 | II | 86（93） | 64/66 | Esophageal squamous cell carcinoma | PD-L1 | Durvalumab | 45 | placebo | 41 | Hyperglycemia |
| Pignata, 2023 | Italy | NCT03503786 | II | 125 | 66/65 | Endometrial cancer | PD-L1 | Avelumab | 61 | carboplatin and paclitaxel | 61 | Hyperglycaemia |
| Powles, 2022 | North America, South America, Europe, Asia, and Australia. | NCT03142334 | Ⅲ | 994(71.03) | 60 | Clear cell renal cell carcinoma | PD-1 | Pembrolizumab | 488 | Placebo | 496 | T1DM; Hyperglycaemia;DKA |
| Reck, 2021 | USA | NCT02142738 | Ⅲ | 305(61.31) | 64.6/66 | NSCLC | PD-1 | Pembrolizumab | 154 | Chemotherapy | 150 | T1DM |
| Ribas, 2015 | 12 countries | NCT01704287 | II | 540(60.56) | 62/60/63 | Melanoma | PD-1 | Pembrolizumab | 178 179 | Chemotherapy | 171 | T1DM |
| Rodríguez-Abreu, 2021 | USA | NCT02578680 | Ⅲ | 616(58.93) | 65/63.5 | NSCLC | PD-1 | Pembrolizumab | 405 | Placebo plus chemotherapy | 202 | T1DM |
| Shitara, 2018 | 30 countries | NCT02370498 | Ⅲ | 592(69.26) | 62.5/60 | Gastric or gastro-oesophageal junction cancer | PD-1 | Pembrolizumab | 294 | Paclitaxel | 276 | T1DM |
| Tsujikawa, 2020 | USA | NCT02243371 | II | 93(65.59) | 64 | Pancreatic Cancer | PD-1 | Nivolumab | 51 | Cy/GVAX & CRS-207 | 42 | Hyperglycemia |
| Usmani, 2019 | 15 countries | NCT02579863 | Ⅲ | 301(46.84) | 74 | Multiple myeloma | PD-1 | Pembrolizumab | 149 | lenalidomide + dexamethasone | 145 | T1DM |
| Wang, 2023 | China | NCT03856411 | Ⅲ | 465(81.08) | 63/61 | NSCLC | PD-1 | Toripalimab | 308 | Placebo+ Chemotherapy | 156 | Hyperglycemia |
| Winer, 2021 | 31 countries | NCT02555657 | Ⅲ | 622(0.32) | 50/53 | Breast cancer | PD-1 | Pembrolizumab | 309 | Chemotherapy | 292 | T1DM |
| Zhou（1）, 2022 | China | NCT03789604 | Ⅲ | 479(79.96) | 62/64 | NSCLC | PD-L1 | Sugemalimab | 320 | Placebo + chemotherapy | 159 | Hyperglycaemia, T2DM, DM |
| Zhou, 2023 | 10 countries | NCT03358875 | Ⅲ | 805(77.27) | 61 | NSCLC | PD-1 | Tislelizumab | 534 | Docetaxel | 258 | Hyperglycemia |
| Abbreviations: ICPis, Immune checkpoint inhibitors; T1DM, Type 1 diabetes mellitus; DKA, Diabetic ketoacidosis; T2DM, Type 2 diabetes; DM, diabetes mellitus | | | | | | | | | | | | |
|  |  |  |  |  |  |  |  |  |  |  |  |  |
