# Supplementary material for: Islet function impairment outcomes of immune checkpoint inhibitors in cancer patients: a systematic review and meta-analysis
Source: Front Immunol. 2026 Mar 19;17:1669492. doi: 10.3389/fimmu.2026.1669492 (PMC13044012; doi:10.3389/fimmu.2026.1669492)
Supplement: Supplementary file 4 [file Table4.docx]

| **Table4. GRADE Assessment** | | | | | | | | | | |
| --- | --- | --- | --- | --- | --- | --- | --- | --- | --- | --- |
| **Outcomes** | **Study Design** | **Risk of Bias** | **Inconsistency** | **Indirectness** | **Imprecision** | **Other Considerations** | **Effect (95% CI)** | I2 | **Quality** | **Importance** |
| T1DM | 17RCTs | very serious | no serious inconsistency | no serious indirectness | no serious imprecision | none | RR 3.38 (1.66 to 6.88) | 0.0% | LOW | CRITICAL |
| DKA | 3RCTs | serious | no serious inconsistency | no serious indirectness | serious | none | RR 4.46 (0.88 to 22.73) | 0.0% | LOW | CRITICAL |
| Hyperglycemia | 15RCTs | very serious | no serious inconsistency | no serious indirectness | serious | none | RR 1.15 (0.97 to 1.37) | 0.0% | VERY LOW | CRITICAL |
| DM | 3RCTs | very serious | no serious inconsistency | no serious indirectness | serious | none | RR 1.23 (0.29 to 5.26) | 0.0% | VERY LOW | CRITICAL |
| IFI at any grade | 31RCTs | serious | no serious inconsistency | no serious indirectness | no serious imprecision | none | RR 1.3 (1.1 to 1.53) | 0.0% | MODERATE | CRITICAL |
| IFI at grade 1-2 | 31RCTs | serious | no serious inconsistency | no serious indirectness | serious | none | RR 1.11 (0.92 to 1.33) | 0.0% | LOW | IMPORTANT |
| IFI at grade 3-5 | 31RCTs | serious | no serious inconsistency | no serious indirectness | no serious imprecision | none | RR 2.2 (1.5 to 3.23) | 0.0% | MODERATE | CRITICAL |
| IFI at any grade of PD-L1 | 8RCTs | serious | no serious inconsistency | no serious indirectness | serious | none | RR 1.07 (0.84 to 1.36) | 9.0% | LOW | IMPORTANT |
| IFI at any grade of PD-1 | 23RCTs | serious | no serious inconsistency | no serious indirectness | no serious imprecision | none | RR 1.53 (1.21 to 1.93) | 0.0% | MODERATE | IMPORTANT |
| IFI at any grade of Durvalumab | 4RCTs | very serious | no serious inconsistency | no serious indirectness | serious | none | RR 0.92 (0.65 to 1.3 | 18.0% | VERY LOW | IMPORTANT |
| IFI at any grade of Pembrolizumab | 15RCTs | serious | no serious inconsistency | no serious indirectness | no serious imprecision | none | RR 2.38 (1.43 to 3.97) | 0.0% | MODERATE | IMPORTANT |
| IFI at any grade of Nivolumab | 3RCTs | very serious | no serious inconsistency | no serious indirectness | no serious imprecision | none | RR 3.17 (0.55 to 18.19) | 0.0% | LOW | IMPORTANT |
| IFI at any grade in NSCLC | 8RCTs | serious | no serious inconsistency | no serious indirectness | no serious imprecision | none | RR 1.32 (1.01 to 1.72) | 0.0% | MODERATE | IMPORTANT |
| IFI at any grade in Gastric or gastro-oesophageal junction cancer | 4RCTs | serious | no serious inconsistency | no serious indirectness | serious | none | RR 3.05 (0.74 to 12.58) | 0.0% | LOW | IMPORTANT |
| IFI at any grade in melanoma | 3RCTs | serious | no serious inconsistency | no serious indirectness | serious | none | RR 7.94 (0.99 to 63.48) | 0.0% | LOW | IMPORTANT |
| IFI at any grade in Ovarian cancer | 3RCTs | serious | no serious inconsistency | no serious indirectness | serious | none | RR 1.07 (0.76 to 1.51) | 0.0% | LOW | IMPORTANT |
| IFI at any grade in breast cancer | 2RCTs | very serious | no serious inconsistency | no serious indirectness | serious | none | RR 0.78 (0.54 to 1.12) | 0.0% | VERY LOW | IMPORTANT |
| IFI at any grade in Urothelial Cancer | 2RCTs | very serious | no serious inconsistency | no serious indirectness | very serious | none | RR 1.36 (0.61 to 3.03) | 0.0% | VERY LOW | IMPORTANT |
| IFI at any grade in SCLC | 2RCTs | serious | no serious inconsistency | no serious indirectness | serious | none | RR 1.97 (0.91 to 4.24) | 0.0% | LOW | IMPORTANT |
| IFI at any grade of ICPis monotherapy | 15RCTs | serious | no serious inconsistency | serious | no serious imprecision | none | RR 1.43 (1.04 to 1.97) | 0.0% | LOW | NOT IMPORTANT |
| IFI at any grade of ICPis combined with chemotherapy | 14RCTs | serious | no serious inconsistency | serious | no serious imprecision | none | RR 1.23 (1.01 to 1.49) | 0.0% | LOW | NOT IMPORTANT |
| IFI at grade3-5 of ICPis monotherapy | 15RCTs | serious | no serious inconsistency | serious | no serious imprecision | none | RR 3.39 (1.79 to 6.43) | 0.0% | LOW | NOT IMPORTANT |
| IFI at grade3-5 of ICPis combined with chemotherapy | 14RCTs | serious | no serious inconsistency | serious | serious | none | RR 1.53 (0.92 to 2.55) | 0.0% | VERY LOW | NOT IMPORTANT |
| RCTs, randomized controlled trials. IFI, Islet function impairment. NSCLC, Non-Small Cell Lung Cancer. SCLC, Small Cell Lung Cancer.T1DM, type 1 diabetes mellitus. ICPis, immune checkpoint inhibitors. DKA, diabetic ketoacidosis. RR, Relative Risk. | | | | | | | | | | |
