# Supplementary material for: Islet function impairment outcomes of immune checkpoint inhibitors in cancer patients: a systematic review and meta-analysis
Source: Front Immunol. 2026 Mar 19;17:1669492. doi: 10.3389/fimmu.2026.1669492 (PMC13044012; doi:10.3389/fimmu.2026.1669492)
Supplement: Supplementary file 5 [file Table5.docx]

Table5. Funnel plot and Sensitivity analysis


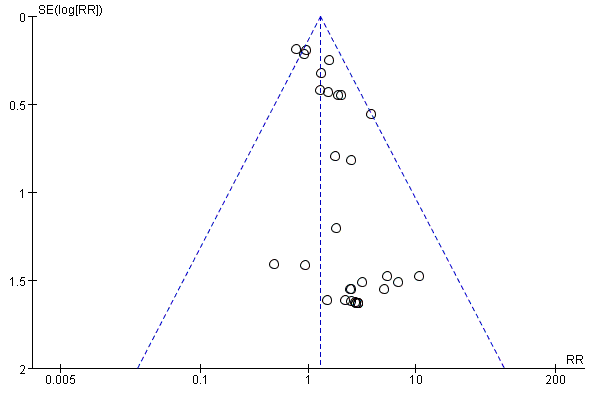


**S1 Fig. Results of funnel plot for the incidence of IFI at any grade of ICPis experimental vs. non-ICPis control**

**
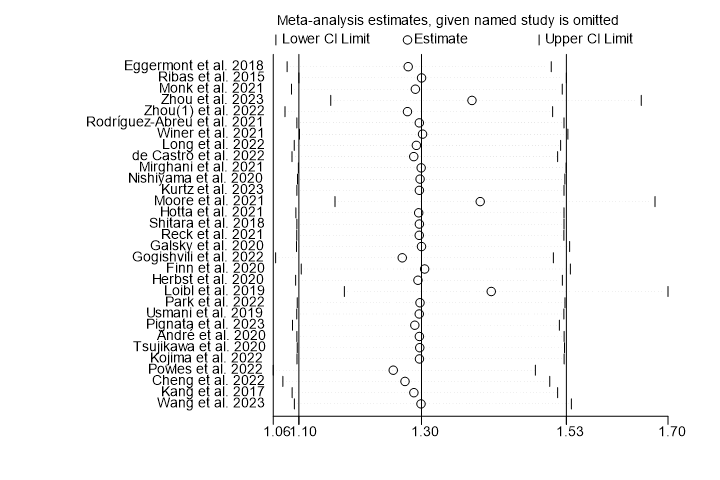
**

**S2 Fig. Results of sensitivity analysis for the incidence of IFI at any grade of ICPis experimental vs. non-ICPis control**

**
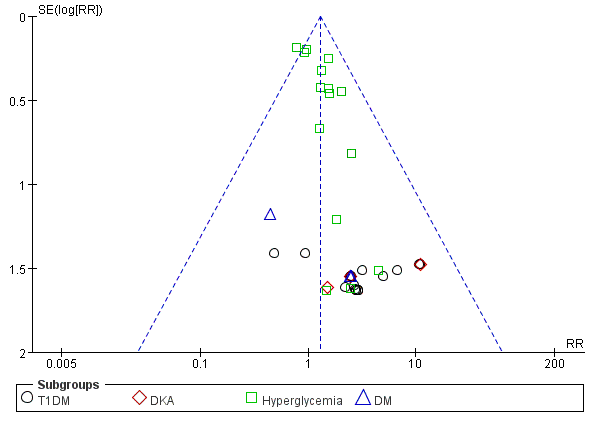
**

**S3 Fig. Results of funnel plot for the incidence of T1DM / DKA / Hyperglycemia of ICPis experimental vs. non-ICPis control**

**
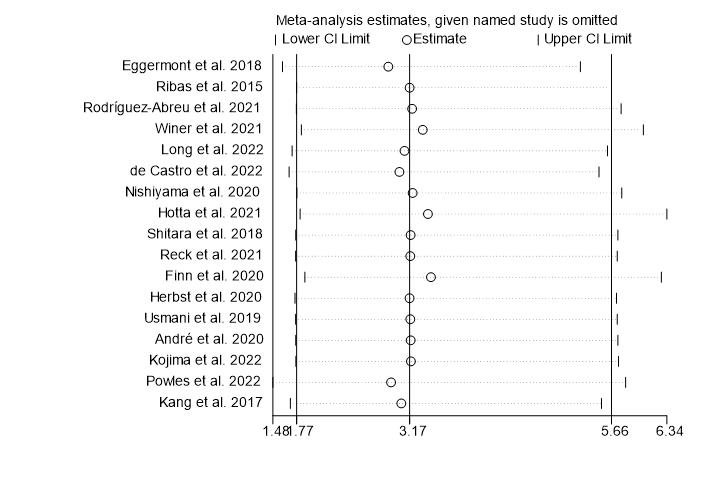
**

**S4 Fig. Results of sensitivity analysis for the incidence of T1DM of ICPis experimental vs. non-ICPis control**

**
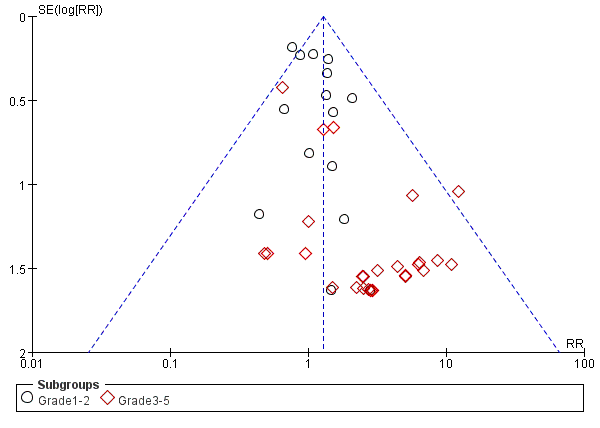
**

**S5 Fig. Results of funnel plot for the incidence of IFI at grade1-2 or grade3-5 of ICPis experimental vs. non-ICPis control**

**
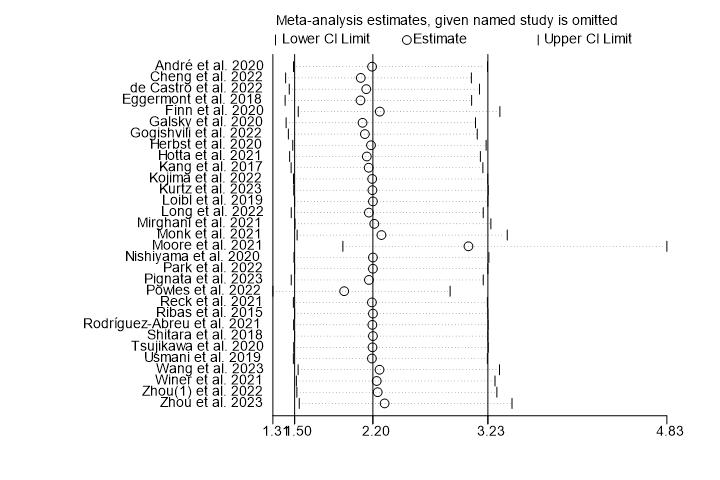
**

**S6 Fig. Results of sensitivity analysis for the incidence of IFI at grade3-5 of ICPis experimental vs. non-ICPis control**

**
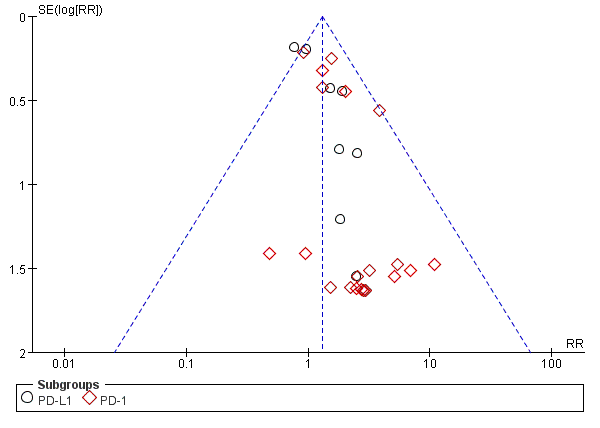
**

**S7 Fig. Results of funnel plot for the incidence of IFI at any grade of PD-L1/PD-1 experimental vs. non- ICPis control**

**
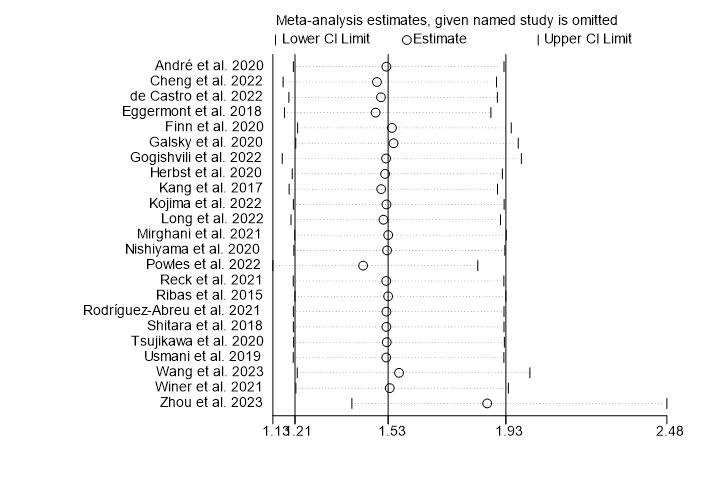
**

**S8 Fig. Results of sensitivity analysis for the incidence of IFI at any grade of PD-1 experimental vs. non- ICPis control**

**
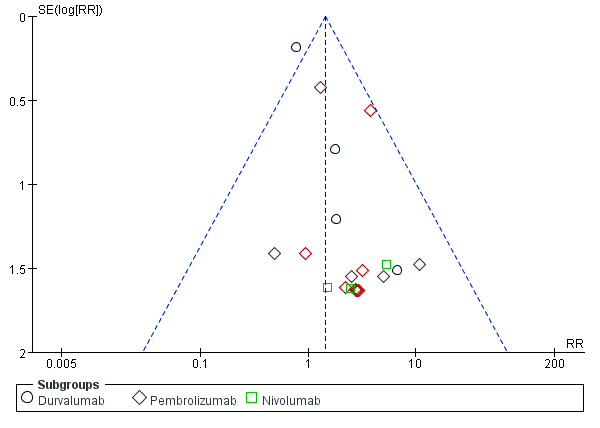
**

**S9 Fig. Results of funnel plot for the incidence of IFI at any grade of Durvalumab/Pembrolizumab/Nivolumab experimental vs. non- ICPis control**

**
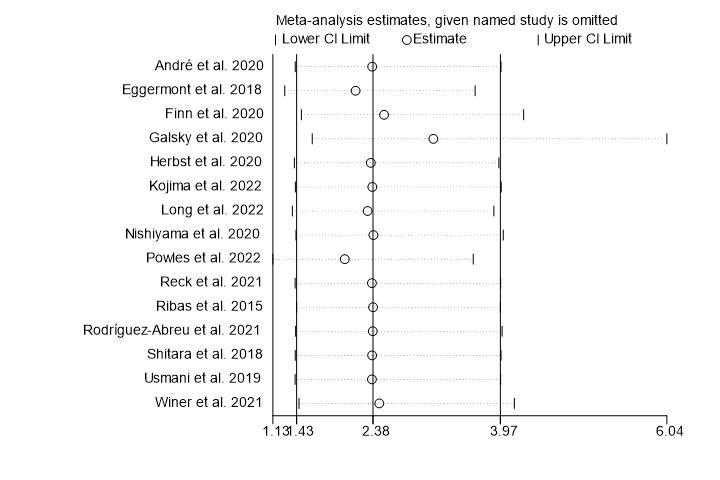
**

**S10 Fig. Results of sensitivity analysis for the incidence of IFI at any grade of Pembrolizumab experimental vs. non- ICPis control**

**
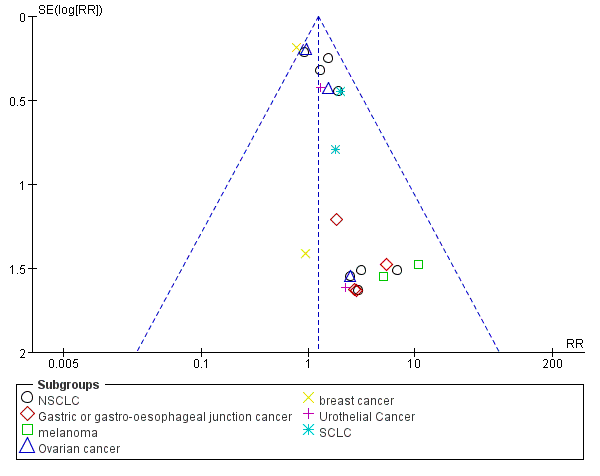
**

**S11 Fig. Results of funnel plot for the incidence of IFI at any grade of ICPis vs. non-ICPis in different tumors**

**
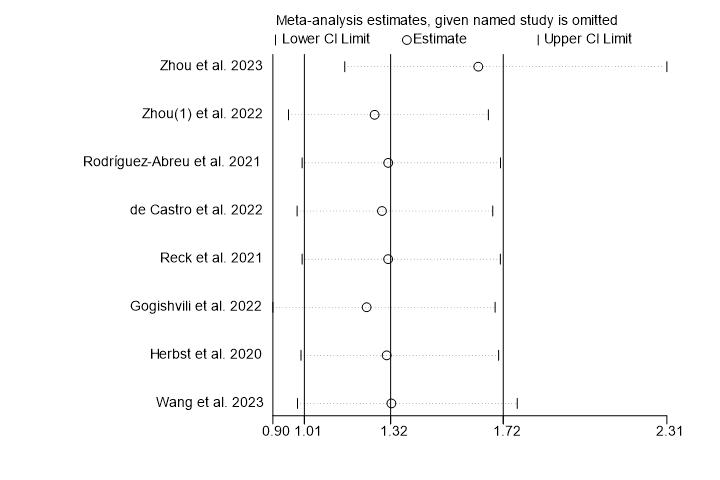
**

**S12 Fig. Results of sensitivity analysis for the incidence of IFI at any grade of ICPis vs. non-ICPis in NSCLC**

**
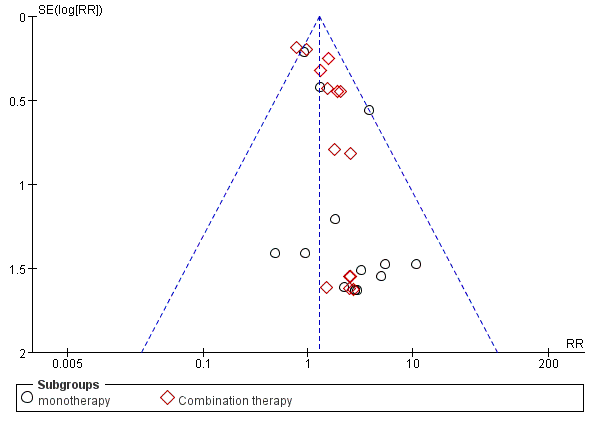
**

**S13 Fig. Results of funnel plot for the incidence of IFI at any grade of ICPis monotherapy / ICPis combined with chemotherapy experimental vs. non-ICPis control**

**
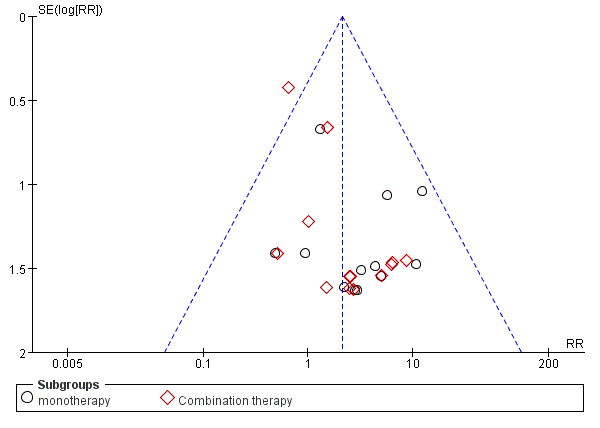
**

**S14 Fig. Results of funnel plot for the incidence of IFI at grade3-5 of ICPis monotherapy /ICPis combined with chemotherapy experimental vs. non-ICPis control**

**
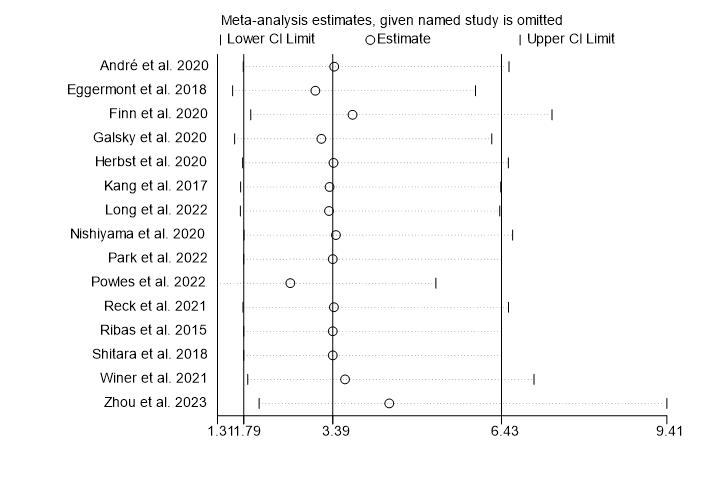
**

**S15 Fig. Results of sensitivity analysis for the incidence of IFI at grade3-5 of ICPis monotherapy /ICPis combined with chemotherapy experimental vs. non-ICPis control**
